# Cardiorespiratory fitness and BMI measured in youth and 5‐year mortality after site‐specific cancer diagnoses in men—A population‐based cohort study with register linkage

**DOI:** 10.1002/cam4.6553

**Published:** 2023-09-21

**Authors:** Aron Onerup, Kirsten Mehlig, Elin Ekblom‐Bak, Lauren Lissner, Mats Börjesson, Maria Åberg

**Affiliations:** ^1^ Department of Pediatrics, Institute of Clinical Sciences, Sahlgrenska Academy University of Gothenburg Gothenburg Sweden; ^2^ Department of Pediatric Oncology, Region Västra Götaland Sahlgrenska University Hospital Gothenburg Sweden; ^3^ School of Public Health and Community Medicine, Institute of Medicine University of Gothenburg Gothenburg Sweden; ^4^ Department of Physical activity and Health The Swedish School of Sport and Health Sciences Stockholm Sweden; ^5^ Department of Molecular and Clinical Medicine, Center for Lifestyle Intervention, Sahlgrenska Academy Gothenburg University Gothenburg Sweden; ^6^ Department of MGAÖ Region of Västra Götaland, Sahlgrenska University Hospital Gothenburg Sweden; ^7^ Region Västra Götaland, Regionhälsan Gothenburg Sweden

**Keywords:** body mass index, epidemiology and prevention, physical activity, prognostic factor, survival

## Abstract

**Background:**

Our aim was to assess associations between cardiorespiratory fitness (CRF) and body mass index (BMI) in youth and 5‐year mortality after site‐specific cancer diagnoses in men.

**Methods:**

Men with cancer from a population who underwent military conscription at ages 16–25 during 1968–2005 in Sweden were included. CRF was assessed as maximal aerobic workload on a cycle ergometer test and was classified as low, moderate, or high. BMI (kg/m^2^) was classified as underweight (<18.5), normal weight (18.5–24.9), overweight (25–29.9), or obesity (>30). Conscription data were linked with register data on cancer diagnosis and mortality. Analyses included CRF, BMI, date of diagnosis, and age, year, and center for conscription.

**Results:**

A total of 84,621 cancer cases were included. Mean age at diagnosis was 52 years. Follow‐up data were available during a mean of 6.5 years. There were linear protective associations between CRF and mortality after any cancer diagnosis (hazard ratio [HR] for high vs. low CRF 0.70), malignant skin cancer (HR 0.80), non‐Hodgkin lymphoma (HR 0.78), and cancer in the lungs (HR 0.80), head and neck (HR 0.68), pancreas (HR 0.83), stomach (HR 0.78), liver (HR 0.84), rectum (HR 0.79), and bladder (HR 0.71). Overweight and/or obesity were associated with increased mortality after any cancer (HR for obesity vs. normal weight 1.89), malignant skin cancer (HR 2.03), Hodgkin lymphoma (HR 2.86) and cancer in the head and neck (HR 1.38), thyroid (HR 3.04), rectum (HR 1.53), kidney (HR 1.90), bladder (HR 2.10), and prostate (HR 2.44).

**Conclusion:**

We report dose‐dependent associations between CRF and BMI in youth and mortality after site‐specific cancer diagnoses in men. The associations with mortality could be due to both cancer inhibition and an improved tolerance to withstand cancer treatment. These results strengthen the incentive for public health efforts aimed at establishing a high CRF and normal weight in youth.

## Graphical abstract

Fitness and body mass index was assessed at age 18 in 1.2 million men. Analyses of associations between fitness and fatness in youth and 5‐year mortality after subsequent cancer diagnoses showed a lower mortality after several of the site‐specific cancers with higher fitness and normal body mass index.

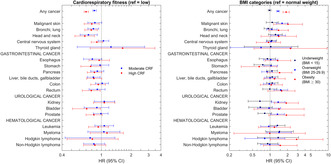

## 1 INTRODUCTION

Cardiorespiratory fitness (CRF) refers to the capacity of the circulatory and respiratory systems to supply oxygen to skeletal muscles during physical activity.^1^ CRF can be improved by physical activity at sufficient intensity and can reflect both an individual's past physical activity as well as his or her ability to be physically active. There are several ways of assessing CRF, but the gold standard is a maximal cardiopulmonary exercise test.^1^ CRF has been reported to have protective associations with several types of cancer.^2^, ^3^ There are a few reports of associations between higher pre‐diagnostic CRF and lower long‐term mortality for individuals diagnosed with any cancer.^4^, ^5^ There are also reports of associations between higher CRF and lower risk of cancer‐specific mortality in the general population.^6^, ^7^, ^8^ A recent systematic review concluded that high CRF was associated with 50% lower mortality among adults diagnosed with any cancer and a 40% lower mortality in adults diagnosed with lung cancer, compared to low CRF.^9^ However, it included cancer survivors followed up for decades after their diagnosis, combining cancer survivorship with cancer mortality. There are reports where self‐reported pre‐ and postdiagnosis level of physical activity (PA) was associated with lower cancer‐specific mortality for breast, colorectal, and prostate cancer.^10^, ^11^, ^12^ CRF is objectively measured and strongly associated with PA of sufficient intensity and has been proposed as an important vital sign to be routinely used in health care.^13^

Higher body mass index (BMI) has been associated with higher risk of developing several site‐specific cancers.^14^ A systematic review and meta‐analysis of the associations between obesity and survival outcomes in patients with cancer with >6 million individuals, reported a 14% higher mortality for any cancer, but a lower mortality after lung cancer, renal cell carcinoma, or melanoma in patients with obesity.^15^ The largest studies included in the review were observational studies analyzing the risk for cancer‐specific mortality in the general population rather than mortality in cancer patients. For several cancer sites, there are reports of an obesity paradox with lower mortality in patients with obesity.^16^, ^17^ There have been speculations on the underlying mechanism, including inflammatory mechanisms from the adipose tissue and poor health status of cancer patients with low BMI, that is, confounding by disease severity.^15^

In summary, there are indications that both CRF and BMI are associated with mortality after any cancer diagnosis and after some site‐specific cancers. However, several of the previous studies mix pre‐ and postdiagnosis assessments, long‐ and short‐term mortality, as well as mortality in patients diagnosed with cancer and cancer‐specific mortality in the general population, and data are lacking for most site‐specific cancers. The potential for improving 5‐year mortality through establishing a healthy lifestyle is still inadequately explored for most cancer sites.^18^, ^19^ Therefore, our aim was to assess the associations between CRF and BMI in youth and the 5‐year mortality following site‐specific cancer diagnosis from an underlying population‐based sample of >1 million men.

## 2 MATERIALS AND METHODS

### 2.1 Design

This is a Swedish nationwide, register‐based observational cohort study with prospective data. Ethical permission for the study was obtained November 16, 2021 from the Swedish authority for ethical permissions, EPN Dnr 462‐14 with addendums Dnr 2021‐05638‐02 and 2023‐04937‐02. No consent was obtained from participants since data were retrieved from registers.

### 2.2 Participants

All men who underwent the compulsory conscription exam from 1968 to 2005 at the age of 16–25 years, with valid information on CRF and BMI and who subsequently were diagnosed with any cancer were included. The World Health Organization defines “youth” as the 15‐ to 24‐year age group.^20^ Men with a cancer diagnosis before or during the same calendar year as the military conscription were excluded.

### 2.3 Data sources

Participants were identified in the Swedish military service conscription register.^21^ The compulsory conscription assessments included measurements of anthropometric measures, blood pressure, muscular strength, and CRF.^22^, ^23^, ^24^, ^25^, ^26^ The Swedish unique personal identification number was used to link conscription data on person‐level with data from Statistics Sweden, the Swedish national patient register,^27^ and the Swedish cause of death register.^28^ The full dataset included information until December 31, 2019.

### 2.4 Exposures

#### 2.4.1 Cardiorespiratory fitness

Information on CRF at conscription was assessed as maximal aerobic workload in units of Watt from a cycle ergometer test (W_max_), as described previously.^21^ Assessments utilized two test procedures with slightly different methods for assessing CRF during the period when assessments were performed.^21^ Results were transformed during conscription to a standardized score (range 1–9),^21^ and CRF was also categorized into low CRF (1–5), moderate CRF (6, 7), and high CRF (8, 9).^23^

#### 2.4.2 Body mass index

Height and weight were measured, and BMI was calculated as kg/m^2^ and categorized into underweight (<18.5 kg/m^2^), normal weight (18.5–24.9 kg/m^2^), overweight (25–29.9 kg/m^2^), and obesity (≥30 kg/m^2^).

### 2.5 Outcomes

#### 2.5.1 Cancer diagnosis

Information on cancer diagnoses was collected from the Swedish National Patient Register^27^ and the cause of death register.^28^ Sweden has a tax‐funded universal health insurance for the entire population and cancer is treated at publicly funded hospitals. Eighteen types of site‐specific cancers as well as any cancer were defined according to ICD8/9/10 codes (Table S1). The first timepoint for a diagnosis was used as diagnosis date. Different site‐specific cancers were treated independently, and an individual could contribute with information on more than one site‐specific cancer. We performed analyses on all cases, as well as analyses restricted to the first cancer diagnosis in each individual. The registers used in this study do not include information on cancer stage or treatment.

#### 2.5.2 Death

Information on date of death was retrieved from the Swedish cause of death register.^28^ With complete coverage at the cause of death register, there is no loss to follow‐up in this study.

### 2.6 Covariates

#### 2.6.1 Muscle strength

Two test procedures were used for muscle strength, previously described in detail.^21^ Muscle strength was categorized into low:1–3, moderate:4–6, and high:7–9.

#### 2.6.2 Parental level of education

Parental level of education was used as socioeconomic status and was collected from Statistics Sweden and categorized according to highest level attained by any parent: up to 9 years of compulsory school, high school ≤2 years at university, or ≥3 years at university.

#### 2.6.3 Smoking habits

For most individuals in this study, there was no information on smoking status. In 1968–1970, questions on smoking were included in the conscription and categorized in our analyses: No active smoking, 1–10 cigarettes or equivalent per day, and > 10 cigarettes or equivalent per day.

### 2.7 Statistical analysis

A statistical analysis plan was specified before any statistical analyses were performed (Supporting Information). No power analysis was performed since the analysis was performed in an existing large population‐based cohort and it has been proposed to refrain from power analyses in such analyses.^29^

Cox proportional hazards models were used to assess the associations between CRF and BMI in youth and the 5‐year mortality following site‐specific cancer diagnoses. Follow‐up started at date of cancer diagnosis until date of death and was censored at first emigration, end of follow‐up (December 31, 2019), and 5 years after diagnosis. The outcome was any mortality. The primary analyses for CRF assessed linear associations between the standardized CRF score (1–9), and supplementary analyses were explored with categorical comparisons (moderate and high vs. low CRF) to facilitate interpretation of the effect sizes. For BMI, the primary analysis was done for categorical comparisons (underweight, overweight, and obesity vs. normal weight) since a U‐shaped trend was anticipated with respect to mortality. The proportional hazards assumption was checked graphically, and no major deviations were observed. Results were given in terms of hazard ratios (HR) with 95% confidence intervals (CI). The statistical analysis plan detailed the covariates to be included in the models, according to a directed acyclic graph. The main analyses included date of cancer diagnosis, the following covariates assessed at the time of conscription: year, age, conscription center, and CRF and BMI. Missing data led to listwise deletion since rate of missing information was low for all included covariates. A sensitivity analysis was performed in a subpopulation with information on smoking to see how adjusting for smoking changed the estimates.

After performing the first analysis, we identified the need to include date of cancer diagnosis to account for changes in cancer outcomes over time. Combined with year of conscription, this effectively also adjusts for age at diagnosis. The pre‐specified analysis was to be performed for all site‐specific cancers regardless of other previous cancers in the individual. After performing the pre‐specified analyses, we identified the need to add an analysis restricted to the first cancer diagnosis in each participant. Sensitivity analyses were also performed to see whether including parental education, age at diagnosis, or muscle strength as covariates in the models changed the results. Missing values lead to listwise deletion.

## 3 RESULTS

**FIGURE 1:**
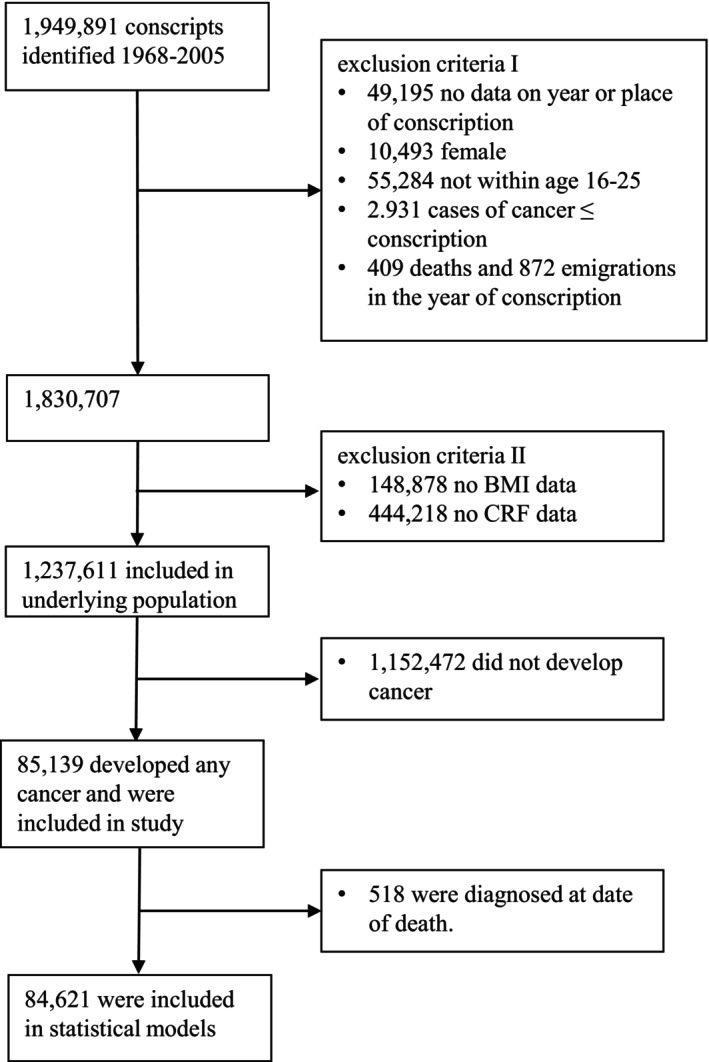
Flow chart of participants included in the study.

From the population of 1,237,611 men, 85,139 were diagnosed with a cancer, of whom 84,621 received their cancer diagnosis before possible date of death (Table 1, Figure 1). Their mean age at diagnosis was 52 years. For men who developed any cancer, proportions/mean values did not differ across CRF categories for age, BMI, blood pressure, and morbidity at conscription (Table 2). Most cancer cases (67%) occurred in men who underwent military conscription in the 1970s, and there was no dramatic change in BMI or CRF during the decades when conscription was performed (Table S2). Men with low CRF were more likely to be underweight, while overweight and obesity were evenly distributed between all CRF groups at conscription. Participants with low CRF were more likely to report smoking and to have parents who had not attained higher education. Follow‐up data were available for a mean of 6.5 years after cancer diagnosis (Table 1). Table 3 shows the main results including all cancer cases while Table 4 shows the same analyses restricted to the first cancer diagnosis in each individual.

**TABLE 1:** Number of men with site‐specific cancers in this population‐based sample of 85,139 cancer cases in Sweden, age at diagnosis and length of follow‐up per cancer site.

Cancer site	Overall			Low CRF		Moderate CRF		High CRF	
	*n* cases	Mean age at diagnosis (SD)	Mean follow‐up, years	*N* (%)	Mean follow‐up, years	*N* (%)	Mean follow‐up, years	*N* (%)	Mean follow‐up, years
Any cancer	85,139	52.0 (10.6)	6.5	28,724 (34%)	6.2	30,917 (36%)	6.5	25,498 (30%)	6.6
Malignant skin	28,418	52.5 (9.1)	6.5	8579 (30%)	6.6	10,350 (36%)	6.4	9489 (33%)	6.4
Bronchi and lung	3083	54.7 (9.2)	2.6	1380 (45%)	2.2	1042 (34%)	3.0	661 (21%)	2.8
Head and neck	3566	51.8 (9.7)	6.8	1365 (38%)	6.5	1253 (35%)	6.9	948 (27%)	7.1
Central nervous system	3282	44.8 (12.2)	5.9	1081 (33%)	5.9	1219 (37%)	5.9	982 (30%)	6.0
Thyroid gland	850	44.6 (11.9)	10.5	271 (32%)	10.1	339 (40%)	10.3	240 (28%)	11.2
Gastrointestinal cancer									
Esophagus	1014	55.1 (8.2)	2.4	437 (43%)	2.2	335 (33%)	2.6	242 (24%)	2.5
Stomach	1299	52.9 (9.3)	2.8	507 (39%)	2.6	461 (35%)	2.8	331 (25%)	3.1
Pancreas	1869	55.2 (8.3)	1.8	696 (37%)	1.7	666 (36%)	1.9	507 (27%)	2.0
Liver, bile ducts and gallbladder	1828	54.7 (9.0)	2.1	791 (43%)	1.8	643 (35%)	2.3	394 (22%)	2.3
Colon	4330	52.8 (9.5)	5.1	1617 (37%)	4.8	1614 (37%)	5.2	1099 (25%)	5.3
Rectum	3149	54.1 (8.4)	4.9	1133 (36%)	4.7	1157 (37%)	4.8	859 (27%)	5.1
Urological cancer									
Kidney	2311	53.1 (9.1)	5.6	880 (38%)	5.6	839 (36%)	5.6	592 (26%)	5.5
Bladder	3087	54.7 (8.8)	6.4	1167 (38%)	6.2	1078 (35%)	6.2	842 (27%)	7.0
Prostate	19,704	58.6 (5.6)	4.6	6773 (34%)	4.6	6756 (34%)	4.7	6175 (31%)	4.6
Hematological cancer									
Leukemia	2832	48.7 (12.1)	6.0	907 (32%)	5.8	1050 (37%)	6.1	875 (31%)	6.2
Myeloma	1217	53.6 (8.8)	5.2	379 (31%)	5.2	442 (36%)	5.2	396 (33%)	5.1
Hodgkin lymphoma	1135	38.5 (11.8)	13.4	354 (31%)	13.1	445 (39%)	12.8	336 (30%	14.6
Non‐Hodgkin lymphoma	3298	49.3 (10.6)	7.4	1065 (32%)	7.3	1252 (38%)	7.4	981 (30%)	7.5

Abbreviation: CRF, cardiorespiratory fitness.

**TABLE 2:** Demographics and baseline characteristics at conscription by CRF level.

	Low^a^ (*N* = 28,724))	Moderate^a^ (*N* = 30,917))	High^a^ (*N* = 25,498)	Overall (*N* = 85,139)
Year for conscription, mean (SD)	1977 (6)	1979 (8)	1977 (7)	1978 (7)
Age at conscription, mean (SD)	18.5 (0.8)	18.4 (0.7)	18.4 (0.6)	18.4 (0.7)
Height, cm, mean (SD)	178 (6)	179 (6)	181 (6)	179 (6)
Body Mass Index (BMI), mean (SD)	20.9 (3.0)	21.5 (2.5)	22.2 (2.5)	21.5 (2.7)
BMI‐category				
Underweight	4985 (17%)	2149 (7%)	490 (2%)	7624 (9%)
Normal weight	21,190 (74%)	26,130 (85%)	22,487 (88%)	69,807 (82%)
Overweight	2086 (7%)	2336 (8%)	2282 (9%)	6704 (8%)
Obesity	463 (2%)	302 (1%)	239 (1%)	1004 (1%)
Systolic blood pressure, mean (SD)	127 (11)	128 (11)	129 (11)	128 (11)
Diastolic blood pressure, mean (SD)	69 (9)	69 (10)	69 (10)	69 (10)
Diabetes mellitus	14 (0.05%)	12 (0.04%)	8 (0.03%)	34 (0.04%
Hypertension	40 (0.1%)	31 (0.1%)	38 (0.2%)	109 (0.1%)
Cardiovascular disease	612 (2%)	625 (2%	552 (2%)	1789 (2%)
Kidney disease	43 (0.2%)	40 (0.1%)	18 (0.1%)	101 (0.1%)
Alcohol abuse	207 (0.7%)	95 (0.3%)	36 (0.14%)	338 (0.4%)
Substance abuse	300 (1.0%)	116 (0.4%)	46 (0.2%)	462 (0.5%)
Parental education				
Compulsory school	9585 (45%)	8945 (37%)	7117 (36%)	25,647 (39%)
High school ≤2 years university	9875 (47%)	12,208 (51%)	9687 (49%)	31,770 (49%)
>2 years university	1762 (8%)	2997 (12%)	3119 (16%)	7878 (12%)
Smoking information 1968–1970	1768	1336	1289	4393
No active smoking	541 (31%)	524 (39%)	683 (53%)	1748 (40%)
Smoking 1–10 cigarettes	588 (33%)	432 (32%)	356 (28%)	1376 (31%)
Smoking >10 cigarettes	597 (34%)	359 (27%)	215 (17%)	1171 (27%)

Abbreviations: BMI, body mass index; CRF, cardiorespiratory fitness.

^a^ Evaluated with maximal aerobic workload and transformed to a standardized score (1–9) and categorized into low CRF (1–5), moderate CRF (6, 7), and high CRF (8, 9). BMI is categorized into underweight (<18.5 kg/m^2^), normal weight (18.5–24.9 kg/m^2^), overweight (25–29.9 kg/m^2^), and obesity (≥30 kg/m^2^). Reference is normal weight.

**TABLE 3:** Associations between cardiorespiratory fitness and body mass index in youth and 5‐year mortality per cancer site for all cancer cases, regardless of previous cancers.

Cancer site	*n* cases	*n* (%) deaths	Cardiorespiratory fitness (ref = low)			BMI (ref = Normal weight)		
			Moderate	High	*p*‐value for linear trend^b^	Underweight	Overweight	Obesity
			HR (95% CI)	HR (95% CI)		HR (95% CI)	HR (95% CI)	HR (95% CI)
Any cancer site	84,621	13,431 (16%)	0.85 (0.82–0.89)	0.70 (0.67–0.74)	<0.001	0.98 (0.93–1.04)	1.37 (1.29–1.45)	1.89 (1.67–2.14)
Malignant skin	28,359	1191 (4%)	0.88 (0.76–1.01)	0.82 (0.71–0.95)	0.001	0.97 (0.80–1.19)	1.51 (1.24–1.83)	1.94 (1.20–3.14)
Bronchi and lung	2502	1706 (68%)	0.83 (0.74–0.93)	0.82 (0.72–0.94)	0.001	0.92 (0.80–1.07)	1.06 (0.87–1.28)	1.17 (0.81–1.69)
Head and neck	3549	715 (20%)	0.84 (0.70–0.99)	0.69 (0.57–0.84)	<0.001	1.06 (0.83–1.36)	1.51 (1.19–1.93)	1.39 (0.74–2.61)
Central nervous system	2937	1566 (53%)	1.05 (0.93–1.19)	0.90 (0.79–1.02)	0.24	1.06 (0.88–1.27)	1.03 (0.86–1.24)	0.93 (0.57–1.50)
Thyroid gland	848	90 (11%)	1.02 (0.61–1.68)	0.94 (0.55–1.61)	0.71	1.16 (0.54–2.48)	1.22 (0.62–2.41)	3.04 (1.22–7.61)
Gastrointestinal cancer								
Esophagus	991	700 (71%)	0.88 (0.74–1.05)	0.93 (0.76–1.13)	0.48	1.00 (0.75–1.32)	1.09 (0.87–1.37)	1.15 (0.75–1.76)
Stomach	1269	791 (62%)	0.98 (0.83–1.16)	0.93 (0.78–1.12)	0.40	0.95 (0.74–1.23)	1.06 (0.85–1.32)	1.23 (0.79–1.91)
Pancreas	1809	1281 (71%)	0.92 (0.81–1.05)	0.83 (0.72–0.96)	0.048	1.07 (0.89–1.29)	0.96 (0.80–1.15)	1.40 (0.90–2.18)
Liver, bile ducts and gallbladder	1573	1062 (68%)	0.85 (0.74–0.98)	0.87 (0.74–1.03)	0.03	1.09 (0.89–1.34)	1.00 (0.82–1.22)	1.17 (0.78–1.76)
Colon	4265	1314 (31%)	1.01 (0.89–1.14)	0.95 (0.82–1.10)	0.33	1.14 (0.94–1.37)	0.99 (0.81–1.19)	1.13 (0.78–1.62)
Rectum	3123	829 (27%)	0.94 (0.80–1.11)	0.82 (0.68–0.98)	0.02	1.00 (0.79–1.28)	1.47 (1.17–1.85)	1.48 (0.91–2.40)
Urological cancer								
Kidney	2288	542 (24%)	1.09 (0.90–1.34)	1.07 (0.86–1.33)	0.51	0.69 (0.48–1.01)	1.36 (1.07–1.73)	1.82 (1.20–2.78)
Bladder	3078	383 (12%)	0.90 (0.71–1.14)	0.72 (0.55–0.94)	0.02	0.75 (0.51–1.10)	0.94 (0.64–1.40)	2.10 (1.11–3.96)
Prostate	19,686	948 (5%)	0.95 (0.82–1.11)	0.83 (0.70–0.98)	0.050	0.84 (0.66–1.06)	1.30 (1.02–1.65)	2.44 (1.41–4.23)
Hematological cancer								
Leukemia	2698	639 (24%)	1.05 (0.86–1.27)	1.00 (0.81–1.23)	0.86	1.39 (1.07–1.81)	0.95 (0.72–1.26)	1.31 (0.75–2.27)
Myeloma	1209	251 (21%)	1.14 (0.83–1.57)	1.20 (0.86–1.66)	0.23	1.65 (1.06–2.58)	1.40 (0.94–2.10)	0.71 (0.17–2.87)
Hodgkin lymphoma	1112	107 (10%)	0.93 (0.59–1.47)	0.81 (0.49–1.35)	0.67	1.55 (0.85–2.84)	0.89 (0.47–1.68)	2.40 (0.95–6.07)
Non‐Hodgkin lymphoma	3261	530 (16%)	0.85 (0.69–1.04)	0.78 (0.63–0.97)	0.01	0.82 (0.58–1.16)	1.14 (0.85–1.51)	1.28 (0.72–2.28)

*Note*: Hazard ratios for mortality in each site‐specific cancer by cardiorespiratory fitness and body composition. Analyses adjusted for year of conscription, conscription center, age at conscription, and date of cancer diagnosis.

Abbreviation: BMI, body mass index.

^a^ Evaluated with maximal aerobic workload and transformed to a standardized score (1–9) and categorized into low CRF (1–5), moderate CRF (6, 7), and high CRF (8, 9), with low being the reference in the analyses.

^b^ Analyzed with the 9‐grade CRF scale. BMI is categorized into underweight (<18.5 kg/m^2^), normal weight (18.5–24.9 kg/m^2^), overweight (25–29.9 kg/m^2^), and obesity (≥30 kg/m^2^). Reference is normal weight.

**TABLE 4:** Associations between cardiorespiratory fitness and body mass index in youth and 5‐year mortality per cancer site restricted to the first cancer diagnosis in each participant.

Cancer site	*n* cases	*n* (%) deaths	Cardiorespiratory fitness (ref = low)			BMI (ref = Normal weight)		
			Moderate	High	*p*‐value for linear trend^a^	Underweight^b^	Overweight^b^	Obesity^b^
			HR (95% CI)	HR (95% CI)		HR (95% CI)	HR (95% CI)	HR (95% CI)
Any cancer site	84,621	13,431 (16%)	0.85 (0.82–0.89)	0.70 (0.67–0.74)	<0.001	0.98 (0.93–1.04)	1.37 (1.29–1.45)	1.89 (1.67–2.14)
Malignant skin	26,401	823 (3%)	0.86 (0.73–1.01)	0.80 (0.67–0.95)	0.002	1.09 (0.86–1.38)	1.61 (1.28–2.02)	2.03 (1.15–3.60)
Bronchi and lung	2141	1416 (66%)	0.83 (0.73–0.94)	0.79 (0.68–0.91)	<0.001	0.93 (0.79–1.09)	1.07 (0.87–1.32)	1.40 (0.95–2.06)
Head and neck	2704	530 (20%)	0.74 (0.61–0.91)	0.68 (0.54–0.85)	<0.001	1.24 (0.94–1.62)	1.38 (1.03–1.84)	1.41 (0.70–2.84)
Central nervous system	2816	1487 (53%)	1.05 (0.93–1.19)	0.90 (0.79–1.03)	0.28	1.07 (0.88–1.29)	1.04 (0.86–1.25)	0.94 (0.58–1.52)
Thyroid gland	712	45 (6%)	1.25 (0.57–2.74)	1.64 (0.77–3.48)	0.13	1.03 (0.36–3.01)	0.70 (0.21–2.35)	2.24 (0.53–9.53)
Gastrointestinal cancer								
Esophagus	695	489 (70%)	0.81 (0.66–1.01)	0.95 (0.75–1.21)	0.23	0.90 (0.65–1.25)	1.03 (0.77–1.36)	1.35 (0.82–2.27)
Stomach	829	518 (62%)	0.97 (0.79–1.19)	0.78 (0.62–0.99)	0.042	0.75 (0.53–1.06)	1.04 (0.79–1.35)	0.65 (0.32–1.31)
Pancreas	1360	972 (71%)	0.91 (0.78–1.06)	0.85 (0.72–1.01)	0.09	1.08 (0.87–1.34)	0.89 (0.72–1.10)	1.46 (0.90–2.37)
Liver, bile ducts and gallbladder	1388	917 (66%)	0.83 (0.72–0.97)	0.84 (0.71–1.01)	0.02	1.09 (0.87–1.37)	1.00 (0.81–1.24)	1.19 (0.78–1.82)
Colon	3440	921 (27%)	1.02 (0.87–1.18)	0.93 (0.78–1.10)	0.23	1.09 (0.87–1.37)	0.97 (0.77–1.21)	1.05 (0.68–1.63)
Rectum	2490	605 (24%)	0.91 (0.75–1.09)	0.79 (0.64–0.97)	0.02	0.99 (0.75–1.31)	1.53 (1.17–2.01)	1.58 (0.92–2.69)
Urological cancer								
Kidney	1877	403 (21%)	1.07 (0.85–1.36)	1.07 (0.83–1.37)	0.41	0.67 (0.42–1.06)	1.38 (1.05–1.81)	1.90 (1.18–3.07)
Bladder	2597	263 (10%)	0.90 (0.67–1.19)	0.71 (0.51–0.98)	0.03	0.68 (0.43–1.09)	0.96 (0.61–1.52)	1.42 (0.53–3.86)
Prostate	18,066	748 (4%)	0.99 (0.83–1.17)	0.84 (0.70–1.02)	0.13	0.81 (0.62–1.06)	1.34 (1.02–1.76)	1.91 (0.95–3.85)
Hematological cancer								
Leukemia	2155	487 (23%)	1.07 (0.86–1.33)	0.93 (0.74–1.18)	0.47	1.34 (1.00–1.81)	0.95 (0.69–1.30)	1.19 (0.63–2.24)
Myeloma	919	176 (19%)	1.06 (0.73–1.54)	1.14 (0.77–1.67)	0.38	1.45 (0.84–2.52)	1.43 (0.88–2.32)	0.99 (0.24–4.04)
Hodgkin lymphoma	873	71 (8%)	0.77 (0.43–1.37)	0.81 (0.45–1.48)	0.80	1.38 (0.61–3.09)	0.81 (0.37–1.80)	2.86 (1.01–8.11)
Non‐Hodgkin lymphoma	2484	364 (15%)	0.84 (0.66–1.08)	0.86 (0.66–1.11)	0.25	0.91 (0.61–1.36)	0.99 (0.69–1.42)	1.50 (0.77–2.92)

*Note*: Hazard rate ratios for mortality in each site‐specific cancer by cardiorespiratory fitness and body composition. Analyses adjusted for year of conscription, conscription center, age at conscription, and date of cancer diagnosis.

Abbreviations: BMI, body mass index; CRF, cardiorespiratory fitness.

^a^ Evaluated with maximal aerobic workload and transformed to a standardized score (1–9) and categorized into low CRF (1–5), moderate CRF (6, 7), and high CRF (8, 9), with low being the reference in the analyses.

^b^ BMI is categorized into underweight (<18.5 kg/m^2^), normal weight (18.5–24.9 kg/m^2^), overweight (25–29.9 kg/m^2^), and obesity (≥30 kg/m^2^). Reference is normal weight.

### 3.1 Any cancer site

**FIGURE 2:**
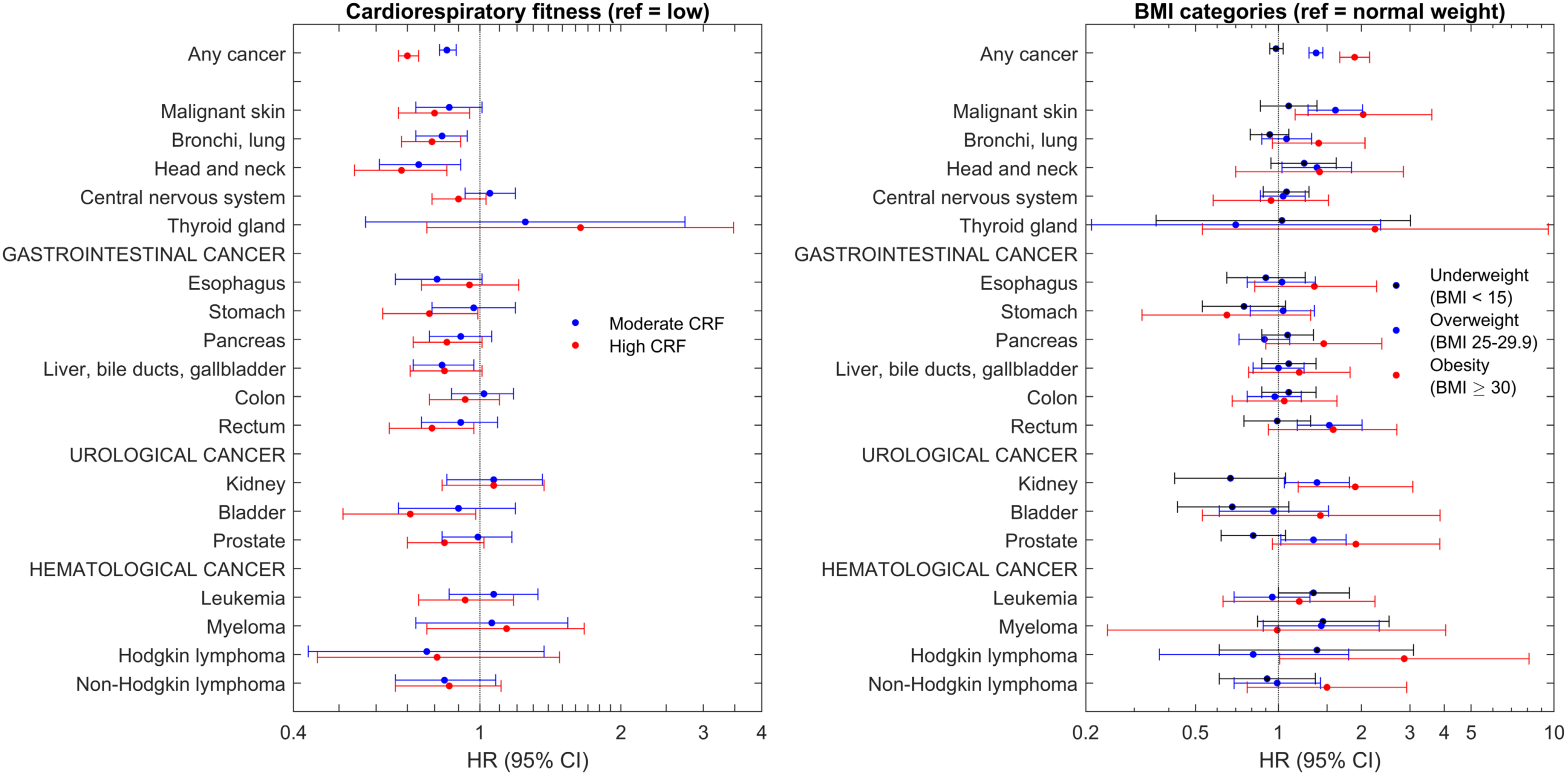
Forest plot illustrating the associations between CRF and BMI and 5‐year mortality restricted to the first cancer in each individual. Numbers provided in Table 4.

There was a linear association between the 9‐grade CRF scale in youth and 5‐year mortality after any cancer diagnosis (*p* < 0.001, hazard ratio [HR] for high vs. low CRF 0.70, 95% confidence interval [CI] 0.67–0.74, Table 3). There was also a dose‐dependent association between BMI and mortality (HR for obesity vs. normal weight 1.89, 95% CI 1.67–2.14, Table 3, Figure 2). Estimates were similar for BMI with and without smoking in the 1968–1970 subsample, while the HR was slightly closer to 1 for CRF when adjusting for smoking (Table S3).

### 3.2 Malignant skin cancer

Higher CRF was linearly associated with lower 5‐year mortality after diagnosis of malignant skin cancer both for all skin cancers (Table 3) and restricted to the first cancer in each individual (*p* = 0.002, HR 0.80, 95% CI 0.67–0.95, Table 4). Overweight and obesity was associated with higher mortality compared to normal weight (HR 2.03, 95% CI 1.15–3.60, Tables 3 and 4). Adjusting for smoking changed the estimates for CRF but estimates for BMI were similar with and without smoking (Table S3).

### 3.3 Bronchi and lungs

There was a linear association between higher CRF and lower 5‐year mortality after lung cancer diagnosis both for any lung cancer and restricted to the first cancer in each individual (*p* < 0.001, HR 0.80, 95% CI 0.67–0.95, Tables 3 and 4). The estimates did not change considerably with and without adjusting for smoking (Table S3). BMI was not associated with mortality after lung cancer diagnosis (HR 1.40, 95% CI 0.95–2.06).

### 3.4 Head and neck

The 5‐year mortality after head and neck cancer diagnosis was linearly associated with lower CRF (*p* < 0.001, HR 0.68, 95% CI 0.54–0.85, Tables 3 and 4). Overweight was associated with an increased mortality (HR 1.38, 95% CI 1.03–1.84), while there was no significant risk increase for obesity (HR 1.41, 95% CI 0.70–2.84, Tables 3 and 4). Models did not converge for the sensitivity analysis of smoking due to too few events.

### 3.5 The central nervous system

There were no associations between CRF (*p* = 0.28, HR 0.90, 95% CI 0.79–1.03) or BMI (HR 0.94, 95% CI 0.58–1.52) and 5‐year mortality after CNS cancer diagnosis (Tables 3 and 4).

### 3.6 Thyroid cancer

There was no association between CRF and mortality after thyroid cancer diagnosis (*p* = 0.13, HR 1.64, 95% CI 0.77–3.48). However, obesity was associated with a threefold increased mortality when including all thyroid cancers (HR 3.04, 95% CI 1.22–7.61, Table 3) but not when restricting only to thyroid cancers representing the first cancer in each individual (HR 2.24, 95% CI 0.53–9.53, Table 4).

### 3.7 The gastrointestinal (GI) tract

There were linear associations with lower 5‐year mortality with higher CRF for cancer in the pancreas (*p* = 0.048, HR 0.83, 95% CI 0.72–0.96, Table 3), stomach (*p* = 0.042, HR 0.78, 95% CI 0.62–0.99, Table 4), liver (*p* = 0.02, HR 0.84, 95% CI 0.71–1.01, Table 4), and rectum (*p* = 0.02, HR 0.79, 95% CI 0.64–0.97, Table 4). The only GI cancer site where mortality was associated with BMI was rectal cancer, where overweight (HR 1.53, 95% CI 1.17–2.01) and obesity (HR 1.58, 95% CI 0.92–2.69) were associated with approximately 50% higher HR, only significant for overweight (Table 4). The effect sizes did not change considerably with and without adjusting for smoking except for rectal cancer where the results were hard to interpret (Table S3).

### 3.8 Urological cancer

Higher CRF was linearly associated with lower 5‐year mortality after a bladder cancer diagnosis (*p* = 0.03, HR 0.71, 95% CI 0.51–0.98), while there was no association between CRF and mortality after kidney (*p* = 0.41, HR 1.07, 95% CI 0.83–1.37, Table 4) or prostate cancer diagnoses (*p* = 0.13, HR 0.84, 95% CI 0.70–1.02, Table 4). Obesity was associated with higher 5‐year mortality after a kidney cancer diagnosis (HR 1.90, 95% CI 1.18–3.07, Table 4) and for bladder (HR 2.10, 95% CI 1.11–3.96), and prostate (HR 2.44, 95% CI 1.41–4.23) cancer diagnoses when including all diagnoses in each individual (Table 3) but not when restricting to the first cancer in each individual (Table 4). Analyses could not be performed in the 1968–1970 smoking population (Table S3).

### 3.9 Hematological malignancies

High CRF was linearly associated with lower 5‐year mortality after non‐Hodgkin lymphoma diagnosis when including both first and second cancers (*p* = 0.01, HR 0.78, 95% CI 0.63–0.97, Table 3) but not when restricted to the first cancer in each individual (*p* = 0.25, HR 0.86, 95% CI 0.66–1.11, Table 4). CRF was not associated with 5‐year mortality after being diagnosed with leukemia (*p* = 0.47, HR 0.93, 95% CI 0.74–1.18, Table 4), myeloma (*p* = 0.38, HR 1.14, 95% CI 0.77–1.67, Table 4), or Hodgkin lymphoma (*p* = 0.80, HR 0.81, 95% CI 0.45–1.48, Table 4). Obesity was associated with higher mortality after a Hodgkin lymphoma diagnosis when restricted to the first cancer in each individual (HR 2.86, 95% CI 1.01–8.11, Table 4) but not when including all Hodgkin lymphoma diagnoses (HR 2.40, 95% CI 0.95–6.07, Table 3). Overweight or obesity were not associated with 5‐year mortality after leukemia (HR 1.19, 95% CI 0.63–2.24), myeloma (HR 0.99, 95% CI 0.24–4.04), or non‐Hodgkin lymphoma (HR 1.50, 95% CI 0.77–2.92) diagnoses (Table 4). However, underweight was associated with higher 5‐year mortality after a leukemia diagnosis (HR 1.34, 95% CI 1.00–1.81, Table 4). The results for leukemia were not confounded by smoking while only leukemia could be analyzed (Table S3).

### 3.10 5‐year mortality after first cancer diagnoses

Restricting the analyses to the first cancer in each individual had little effect on most of the results (Table 4). For CRF, it was now linearly associated with mortality after stomach cancer diagnosis, while the linear association between CRF and mortality after non‐Hodgkin lymphoma diagnosis was no longer significant, suggesting confounding by previous cancer. The linear association with mortality after pancreas cancer was no longer significant while the estimates for the categorical comparisons for pancreas cancer were unchanged, suggesting a lack of power rather than confounding. For BMI, the risk increases associated with obesity were reduced for mortality after cancer diagnoses in the thyroid, bladder, and prostate, and were no longer significant, indicating partial confounding by previous cancers for these sites (Table 4). Conversely, obesity was now associated with a considerable increased mortality after Hodgkin lymphoma diagnosis.

### 3.11 10‐ and 15‐year mortality

The analyses for 5‐, 10‐, and 15‐year mortality showed consistent results for most site‐specific cancers (Table S4). For the hematological malignancies where underweight was associated with increased mortality, the risk increases disappeared over time, indicating a cancer‐specific increased mortality. For CRF and obesity, the effect sizes were generally consistent with increasing follow‐up time.

### 3.12 Further sensitivity analyses

Including parental level of education as a marker of socioeconomic status or muscle strength did not change the *p*‐values for trends or the effect sizes (Tables S5 and S6). While there were some cancer sites where the estimates differed by time period, for example, myeloma, the association between CRF and 5‐year mortality was relatively consistent regardless of year of conscription (Table S7). This also applied to the associations between BMI and mortality (Table S8). Both sets of analyses were limited by wide confidence intervals for the later time period due to few cancer diagnoses in men who underwent conscription after 1979. Tables S9 and S10 illustrate analyses stratified by BMI and CRF respectively to further assess the obesity paradox. While the estimates differed slightly for most cancer sites, the association between BMI and mortality did not change direction between men with low versus moderate‐high CRF (Table S10). This was also true for the association between CRF and mortality, where the hazard was consistent across BMI strata for all cancer sites except for some hematologic malignancies, possibly caused by wide confidence intervals due to few cases (Table S9).

## 4 DISCUSSION

The results from this large population‐based cohort study expands the current knowledge on the associations between CRF and BMI in youth and 5‐year mortality after site‐specific cancer diagnoses. We can confirm previous reports of lower mortality after developing any cancer and lung cancer, for those with higher CRF.^4^, ^5^, ^9^ In addition, to our knowledge, our study is the first to report associations between higher CRF and a lower 5‐year mortality after malignant skin cancer, non‐Hodgkin lymphoma, and cancer in the head and neck, pancreas, liver, rectum, bladder, and prostate. We can also confirm previous reports of associations between obesity and higher mortality after any cancer,^30^ and further report a higher 5‐year mortality after malignant skin cancer and cancer in the head and neck, rectum, bladder, prostate, and thyroid for those with obesity and a higher mortality after leukemia and myeloma for those with underweight.

### 4.1 Results for CRF in relation to previous studies

Our study reports linear associations with lower 5‐year mortality for those with higher CRF after diagnosis of any cancer, malignant skin cancer, non‐Hodgkin lymphoma, and cancer in the lung, head and neck, pancreas, liver, rectum, bladder, and prostate. Fardman et al. reported 26% lower mortality after any cancer diagnosis for those with high midlife CRF,^4^ while Lakoski et al. reported 32% lower cancer‐related mortality and 68% lower cardiovascular‐related mortality after any cancer for those with high midlife CRF.^5^ This is well in line with our results with a 30% lower 5‐year mortality for those with high CRF in youth compared to those with low CRF. Our results are also in line with previous studies showing inverse associations between CRF and premature mortality.^31^ For the site‐specific cancers where our study showed linear associations, the relative reductions in mortality were 20%–30% and to our knowledge these are novel findings. Some of the associations between CRF and mortality in the current study were seen for cancer sites with high 5‐year mortality, for example, the lungs and bronchi and pancreas. The 17% relative reductions combined with 70% 5‐year mortality would translate to 12% absolute reductions in 5‐year mortality. If these results could be repeated in public health interventions aiming to increase CRF in the general population, it could be an additional benefit to standard therapy in line with new and expensive medications, from an intervention which is cheap, free from adverse events and with several other health benefits. However, all individuals in this study received conventional care and our results should not be interpreted as support for replacing conventional antitumoral therapy with fitness exercise or weight management.

### 4.2 Results for BMI in relation to previous studies

Silventoinen et al. performed a similar study on information derived from the same registries and reported a 68% higher mortality in men with obesity following a cancer diagnosis, compared to normal weight.^30^ However, they used a smaller underlying population and a shorter follow‐up as well as no restriction in follow‐up after a cancer diagnosis. This could explain why our HR was 92% higher for individuals with obesity versus normal weight. We are not aware of any studies on associations between pre‐diagnostic BMI and 5‐year mortality after site‐specific cancers. However, for several cancer sites, there are reports of an obesity paradox with lower mortality in patients with obesity.^15^ There have been several speculations on the underlying mechanism for that, including inflammatory mechanisms from the adipose tissue and poor health status of cancer patients with low BMI, that is, confounding by disease severity. Interestingly, there were very few signs of this paradox in our study. While studies have reported this mainly for lung cancer and renal cell carcinomas,^15^ our study showed a significantly higher 5‐year mortality in individuals with obesity and kidney cancer and no association between overweight/obesity and mortality after lung cancer. This could indicate that confounding by disease severity was a contributor to the previous studies since that is not present in our study. However, another explanation may be the type of mortality. A study on the obesity paradox in renal cell carcinoma patients reported higher cancer‐specific, but lower overall survival in patients with overweight and obesity.^32^ This corresponds well to our results, where we analyze overall mortality. Our study showed a three times higher mortality after a thyroid cancer diagnosis for men with obesity. This might be explained by the more aggressive clinicopathological features previously reported.^33^ The absence of an increased mortality after liver cancer with increasing BMI is in line with a previous study.^34^ Our stratified analyses also showed that higher CRF was associated with lower mortality regardless of BMI and that higher BMI was a risk factor regardless of CRF.

This study is one of four parallel studies in the same project. In the first study, we assessed the associations between CRF and BMI and the incidence of site‐specific cancers.^2^ We found that higher CRF was associated with lower risk of 9/18 site‐specific cancers. However, higher CRF was also associated with higher risk of being diagnosed with prostate cancer and malignant skin cancer. In the second study, we assessed associations between BMI and site‐specific cancer incidence.^35^ Since the current study showed opposite associations between CRF and mortality after being diagnosed with prostate and skin cancers than our previous study showed for the risk of being diagnosed,^2^ we performed analyses of associations between CRF and BMI and cancer site‐specific mortality in the full study population to see whether high CRF was associated with fatal prostate cancer or skin cancer.^36^ We reported higher risk of dying from skin cancer (HR 1.45, 95% CI 1.16–1.81) but no association between CRF and prostate cancer‐associated mortality (HR 0.95, 95% CI 0.75–1.20). This implies that the results in our current study could be confounded by differences in health seeking behavior leading to higher proportions of low‐risk cancers for prostate cancer and skin cancer, where our previous studies showed unexpected lower risk for men with low CRF and high BMI. However, this explanation does not hold for the other cancer sites.

### 4.3 Strengths and limitations

This study has several strengths. These include the population‐based sample, the large sample size of the underlying population and long follow‐up, resulting in many cancer diagnoses. The validity and full coverage of the population‐based registries in a population covered by universal healthcare insurance increase the validity. The objective assessment of CRF improves sensitivity compared to self‐reported PA, and the inclusion of both BMI and CRF in the analyses improves discrimination between the effects of the underlying lifestyle habits physical activity and diet. The use of the widely used 5‐year mortality as the outcome facilitates comparisons with other risk factors. The assessment of CRF and BMI in youth eliminates the risk of reverse causality by disease severity. However, this also is a limitation since both CRF and BMI may have changed over the time from assessment to cancer diagnosis and we cannot assess associations between CRF and BMI in different parts of life and mortality following a cancer diagnosis. Combined with no repeated assessments during adulthood, our results have implications at a public health level rather than on a clinical level. Our results should be complemented by studies where CRF and BMI were assessed 5 years before the cancer diagnosis. Another limitation in this study is the lack of information on other important risk factors for poor outcome after a cancer diagnosis. We have used the available information on smoking to estimate the confounding effect on mortality, while this was not possible for the site‐specific cancers with low incidence and/or low 5‐year mortality. The results indicate that smoking was not as strong a risk factor for 5‐year mortality as it is for the development of some cancers, and smoking status did not seem to confound our results. Our sensitivity analyses adjusting for parental education, age at diagnosis, and muscle strength did not show any signs of confounding of our results. We lack information on cancer‐specific risk factors, for example, staging, and cancer treatment. There is little reason to believe that CRF or BMI should influence these variables considerably in Sweden where the full population is covered by universal healthcare insurance. However, our previous study showed increased risk with higher CRF and a protective association with increasing BMI for prostate cancer and skin cancer. This might be explained by increased screening, which could result in prostate and skin cancers diagnosed at more beneficial stages,^37^ resulting in better survival. Hence, our current results with lower 5‐year mortality for skin and prostate cancer patients should be interpreted with caution. For all other site‐specific cancers, our previous results show lower risk or same risk of developing site‐specific cancers for those with high CRF and normal weight. Thus, if those with high CRF and normal weight who develop site‐specific cancers have less aggressive cancers, it could be hypothesized that this is yet another of the advantages of having a high CRF and normal weight, rather than a confounding factor. The proportion of men with obesity was low in our population and might explain the significant associations between overweight but not obesity and some cancers. Our study population underwent military conscription during a period of 37 years, which introduces a risk of bias due to changes in distribution of the exposures (CRF and BMI) and improvements in oncology outcomes during this time. However, while the obesity prevalence increased during the study period, the obesity prevalence was still only 2% during the last decade (Table S2) and we have adjusted the analyses for date of cancer diagnosis to account for this. We did not perform any sample size calculation and the large number of associations indicates that the sample size had statistical power to detect associations. However, the number of analyses performed increases the risk of mass significance. Since we considered both BMI and CRF as well as each site‐specific cancer to be of equal interest and to represent its own hypothesis we did not adjust for multiple comparisons. However, this should be considered when interpreting our results. Since this is an observational cohort study, there is always the risk of residual confounding and causality cannot be concluded.

### 4.4 Conclusion

Our study shows that high CRF in youth was associated with 30% lower 5‐year mortality after being diagnosed with any cancer as well as with lower mortality after diagnosis of several site‐specific cancers. Obesity was associated with 89% higher mortality after being diagnosed with any cancer and with higher mortality after being diagnosed with several site‐specific cancers. These results should encourage further promotion of intensified public health work to achieve a high CRF and normal weight early in life.

## AUTHOR CONTRIBUTIONS

**Aron Onerup:** Conceptualization (lead); data curation (equal); formal analysis (lead); funding acquisition (equal); investigation (lead); methodology (lead); project administration (equal); visualization (equal); writing – original draft (lead); writing – review and editing (lead). **Kirsten Mehlig:** Conceptualization (equal); data curation (lead); formal analysis (supporting); investigation (equal); methodology (equal); software (equal); validation (lead); visualization (equal); writing – original draft (supporting); writing – review and editing (equal). **Elin Ekblom‐Bak:** Conceptualization (supporting); investigation (equal); methodology; writing – original draft (supporting); writing – review and editing (equal). **Lauren Lissner:** Conceptualization (supporting); funding acquisition (equal); investigation (equal); methodology (equal); project administration (supporting); supervision (supporting); writing – original draft (supporting); writing – review and editing (equal). **Mats Börjesson:** Conceptualization (supporting); funding acquisition (equal); investigation (equal); methodology (equal); supervision (equal); writing – original draft (supporting); writing – review and editing (equal). **Maria Åberg:** Conceptualization (equal); data curation (supporting); formal analysis (supporting); funding acquisition (equal); investigation (equal); methodology (equal); project administration (supporting); resources (equal); supervision (equal); validation (supporting); writing – original draft (supporting); writing – review and editing (equal).

## FUNDING INFORMATION

This work was supported by grants from the Swedish state under the agreement between the Swedish Government and the county councils, the ALF‐agreement (ALFGBG‐813511, ALFGBG‐965149, ALFGBG‐30411, and ALFGBG‐720691), Assar Gabrielsson's foundation (FB21‐04), the Swedish Research Council (2022‐00166), and the Heart and Lung Foundation (20180379). The funders had no role in planning, interpreting, or reporting the results of the study.

## CONFLICT OF INTEREST STATEMENT

All authors have completed the ICMJE uniform disclosure form at www.icmje.org/coi_disclosure.pdf and declare: EEB, MÅ KM, and LL declare no support from any organization for the submitted work: AO reports grants from Assar Gabrielsson's foundation during the conduct of the study: MB reports grants from the Swedish State under the LUA/ALF agreement and from the Heart and Lung Foundation during the conduct of the study; AO, EEB, MÅ, KM, and MB declare no financial relationships with any organizations that might have an interest in the submitted work in the previous 3 years: LL report grants from the Swedish State under the LUA/ALF agreement and the Swedish Research Council (Vetenskapsrådet) outside the submitted work: LL reports roles in the International Scientific Committee of Choices international, the board of Parker Institute, and the Scientific Advisory Committee of BIPS‐Leibniz Institute; all authors report no other relationships or activities that could appear to have influenced the submitted work. All authors confirm that they had full access to all the data in the study and accept responsibility to submit for publication.

## ETHICS STATEMENT

Ethical permission for the study was obtained 2021‐11‐16 from the Swedish authority for ethical permissions, Dnr 2021–05638‐02 and 2023‐04937‐02.

## CONSENT

No consent was obtained from participants since data were retrieved from registers.

## Supporting information


Data S1.

## Data Availability

The data analyzed in this study are available from the registries used.
