# Supplementary material for: Cardiorespiratory fitness and BMI measured in youth and 5‐year mortality after site‐specific cancer diagnoses in men—A population‐based cohort study with register linkage
Source: Cancer Med. 2023 Sep 21;12(19):20000–14. doi: 10.1002/cam4.6553 (PMC10587926; doi:10.1002/cam4.6553)
Supplement: Supplementary file 1 — Data S1. [file CAM4-12-20000-s001.docx]

Supplementary table S1. Definitions for all site-specific cancers according to ICD diagnosis.

| Type of cancer | ICD-8 | ICD-9 | ICD-10 |
| --- | --- | --- | --- |
| Prostate | 185 | 185 | C61 |
| Bronchi and lung | 162 | 162 | C34 |
| Colon | 153 | 153 | C18 |
| Rectum | 154 | 154 | C19 & C20 |
| Malignant skin | 172-173 | 172-173 | C43-C44 |
| Non-Hodgkin lymphoma | 200 | 200 | C82-C86 |
| Renal | 189 | 189 | C64 |
| Head and neck | 140-149 | 140-149 | C00-C14  C30-C32 |
| Central nervous system | 191-192 | 191-192, 194D & 194E | C70- C72, C75.1-C75.3 |
| Pancreas | 157 | 157 | C25 |
| Bladder | 188 | 188 | C67 |
| Esophagus | 150 | 150 | C15 |
| Stomach | 151 | 151 | C16 |
| Liver, bile ducts and gallbladder | 155-156 | 155-156. Not 155C | C22-C24. Not C22.9. |
| Myeloma | 203 | 203 | C90 |
| Thyroid gland | 193 | 193 | C73 |
| Hodgkin lymphoma | 201 | 201 | C81 |
| Leukemia | 204-207 | 204-208 | C91-C95 |

Supplementary table S2. Distributions for exposures by conscription decade.

|  | 1968-1979  (N=56,751) | 1980-1989  (N=21,811) | 1990-2005  (N=6,577) |  |
| --- | --- | --- | --- | --- |
| CRF level^a^ |  |  |  |  |
| Low | 19,470 (34%) | 7,807 (36%) | 1,447 (22%) |  |
| Moderate | 19,065 (34%) | 8,455 (39%) | 3,397 (52%) |  |
| High | 18,216 (32%) | 5,549 (25%) | 1,733 (26%) |  |
| BMI, mean (SD) | 21.3 (2.6) | 21.8 (2.8) | 22.2 (2.9) |  |
| BMI-category |  |  |  |  |
| Underweight | 5,753 (10%) | 1,547 (7%) | 324 (5%) |  |
| Normal weight | 46,486 (82%) | 18,002 (83%) | 5,319 (81%) |  |
| Overweight | 3,964 (7%) | 1,939 (9%) | 801 (12%) |  |
| Obesity | 548 (1%) | 323 (1%) | 133 (2%) |  |

CRF = Cardiorespiratory fitness. BMI = Body mass index. Categorized into underweight (<18.5 kg/m2), normal weight (18.5-24.9 kg/m2), overweight (25-29.9 kg/m2), and obesity (≥30 kg/m2). Reference is normal weight.

Supplementary table S3. Analyses of five-year mortality performed in subpopulation with information on smoking (n=4,361).

|  |  |  | Cardiorespiratory fitness (ref = low) | BMI (ref = Normal weight) | | Smoking (ref = no smoking) | |
| --- | --- | --- | --- | --- | --- | --- | --- |
| Cancer site | **n** | **Deaths, n (%)** | **Moderate/high** | **Underweight** | **Overweight/obesity** | **1-10 cigarettes per day** | **>10 cigarettes per day** |
|  |  |  | **HRR (95% CI)** | **HRR (95% CI)** | **HRR (95% CI)** | **HRR (95% CI)** | **HRR (95% CI)** |
| Any cancer | 4,361 | 954 (22%) | 0.82 (0.72-0.94) | 0.94 (0.77-1.14) | 1.63 (1.31-2.03) |  |  |
| *Adjusted for smoking* |  |  | 0.89 (0.78-1.02) | 0.93 (0.77-1.14) | 1.62 (1.31-2.02) | 1.42 (1.21-1.68) | 1.92 (1.64-2.26) |
| Malignant skin | 1,225 | 84 (7%) | 0.91 (0.57-1.43) | 0.95 (0.47-1.89) | 1.60 (0.76-3.36) |  |  |
| *Adjusted for smoking* |  |  | 1.03 (0.50-2.02) | 1.01 (0.50-2.02) | 1.60 (0.76-3.36) | 1.21 (0.70-2.10) | 2.35 (1.40-3.96) |
| Bronchi and lung | 239 | 185 (77%) | 1.09 (0.80-1.48) | 0.81 (0.53-1.26) | 1.75 (1.00-3.06) |  |  |
| *Adjusted for smoking* |  |  | 1.11 (0.82-1.50) | 0.76 (0.48-1.18) | 1.74 (0.98-3.09) | 2.14 (1.29-3.57) | 1.67 (1.02-2.74) |
| Esophagus | 80 | 66 (83%) | 0.64 (0.39-1.08) | 1.21 (0.52-2.82) | 0.39 (0.13-1.10) |  |  |
| *Adjusted for smoking* |  |  | 0.68 (0.40-1.15) | 1.26 (0.54-2.97) | 0.36 (0.13-1.03) | 0.80 (0.37-1.70) | 1.20 (0.58-2.50) |
| Colon | 208 | 71 (34%) | 1.36 (0.80-2.32) | 1.48 (0.69-3.15) | 1.09 (0.42-2.79) |  |  |
| *Adjusted for smoking* |  |  | 1.31 (0.76-2.26) | 1.46 (0.69-3.09) | 1.11 (0.42-2.93) | 0.64 (0.37-1.13) | 0.75 (0.40-1.43) |
| Rectum | 155 | 48 (31%) | 0.94 (0.48-1.86) | 0.40 (0.11-1.42) | 1.73 (0.51-5.81) |  |  |
| *Adjusted for smoking* |  |  | 1.15 (0.56-2.37) | 0.44 (0.12-1.55) | 1.72 (0.51-5.80) | 1.13 (0.53-2.41) | 1.83 (0.84-3.99) |
| Pancreas | 113 | 90 (80%) | 0.70 (0.45-1.07) | 1.03 (0.54-1.96) | 1.03 (0.57-1.86) |  |  |
| *Adjusted for smoking* |  |  | 0.68 (0.44-1.05) | 0.99 (0.51-1.90) | 1.08 (0.59-1.97) | 0.91 (0.54-1.52) | 0.64 (0.36-1.13) |
| Leukemia | 132 | 40 (30%) | 0.60 (0.28-1.29) | 1.71 (0.70-4.20) | 2.96 (1.31-6.70) |  |  |
| *Adjusted for smoking* |  |  | 0.59 (0.28-1.28) | 1.66 (0.67-4.09) | 3.11 (1.36-7.11) | 1.17 (0.56-2.43) | 0.87 (0.37-2.07) |

Analyses adjusted for site and year of conscription, age at conscription, and smoking status at conscription. For prostate, stomach, liver, head and neck, kidney, bladder, CNS, thyroid, non-Hodgkin lymphoma, Hodgkin lymphoma, and myeloma, HRR estimates could not be calculated. BMI = Body mass index. Categorized into underweight (<18.5 kg/m2), normal weight (18.5-24.9 kg/m2), overweight (25-29.9 kg/m2), and obesity (≥30 kg/m2). Reference is normal weight.

Supplementary table S4. Five-, ten- and 15-year mortality per cancer site for all cancer cases.

|  |  |  | Cardiorespiratory fitness^a^ (ref = low) | | | BMI (ref = Normal weight) | | |
| --- | --- | --- | --- | --- | --- | --- | --- | --- |
| Cancer site | **n cases** | **n (%) deaths** | **Moderate** | **High** | **p-value for linear trend^b^** | **Underweight** | **Overweight** | **Obesity** |
|  |  |  | **HR (95% CI)** | **HR (95% CI)** |  | **HR (95% CI)** | **HR (95% CI)** | **HR (95% CI)** |
| Any cancer site |  |  |  |  |  |  |  |  |
| *5-year mortality* | 84,621 | 13,431 | 0.85 (0.82-0.89) | 0.70 (0.67-0.74) | <0.001 | 0.98 (0.93-1.04) | 1.37 (1.29-1.45) | 1.89 (1.67-2.14) |
| *10-year mortality* |  | 15,077 | 0.86 (0.83-0.89) | 0.71 (0.68-0.74) | <0.001 | 0.98 (0.92-1.03) | 1.35 (1.28-1.43) | 1.85 (1.64-2.08) |
| *15-year mortality* |  | 15,657 | 0.86 (0.82-0.89) | 0.71 (0.69-0.74) | <0.001 | 0.97 (0.92-1.03) | 1.36 (1.29-1.43) | 1.85 (1.65-2.08) |
| Malignant skin |  |  |  |  |  |  |  |  |
| *5-year mortality* | 28,359 | 1,191 | 0.88 (0.76-1.01) | 0.82 (0.71-0.95) | 0.001 | 0.97 (0.80-1.19) | 1.51 (1.24-1.83) | 1.94 (1.20-3.14) |
| *10-year mortality* |  | 1,548 | 0.90 (0.80-1.02) | 0.83 (0.73-0.95) | 0.001 | 0.97 (0.82-1.16) | 1.50 (1.26-1.78) | 1.86 (1.21-2.86) |
| *15-year mortality* |  | 1,687 | 0.90 (0.80-1.01) | 0.84 (0.74-0.94) | <0.001 | 0.98 (0.83-1.16) | 1.51 (1.28-1.78) | 1.94 (1.29-2.90) |
| Bronchi and lung |  |  |  |  |  |  |  |  |
| *5-year mortality* | 2,502 | 1,706 | 0.83 (0.74-0.93) | 0.82 (0.72-0.94) | 0.001 | 0.92 (0.80-1.07) | 1.06 (0.87-1.28) | 1.17 (0.81-1.69) |
| *10-year mortality* |  | 1,749 | 0.84 (0.75-0.94) | 0.83 (0.73-0.94) | 0.001 | 0.93 (0.81-1.08) | 1.05 (0.87-1.27) | 1.15 (0.80-1.66) |
| *15-year mortality* |  | 1,766 | 0.83 (0.75-0.93) | 0.83 (0.73-0.94) | 0.001 | 0.93 (0.81-1.08) | 1.04 (0.87-1.26) | 1.13 (0.79-1.63) |
| Head and neck |  |  |  |  |  |  |  |  |
| *5-year mortality* | 3,549 | 715 | 0.84 (0.70-0.99) | 0.69 (0.57-0.84) | <0.001 | 1.06 (0.83-1.36) | 1.51 (1.191.93) | 1.39 (0.74-2.61) |
| *10-year mortality* |  | 827 | 0.82 (0.70-0.97) | 0.69 (0.58-0.83) | <0.001 | 1.05 (0.84-1.32) | 1.40 (1.11-1.77) | 1.37 (0.75-2.48) |
| *15-year mortality* |  | 873 | 0.80 (0.69-0.94) | 0.69 (0.58-0.82) | <0.001 | 1.05 (0.84-1.31) | 1.40 (1.11-1.76) | 1.30 (0.71-2.35) |
| Central nervous system |  |  |  |  |  |  |  |  |
| *5-year mortality* | 2,937 | 1,566 | 1.05 (0.93-1.19) | 0.90 (0.79-1.02) | 0.24 | 1.06 (0.88-1.27) | 1.03 (0.86-1.24) | 0.93 (0.57-1.50) |
| *10-year mortality* |  | 1,779 | 1.08 (0.96-1.21) | 0.94 (0.83-1.06) | 0.53 | 1.04 (0.87-1.24) | 1.02 (0.86-1.21) | 0.89 (0.56-1.40) |
| *15-year mortality* |  | 1,857 | 1.06 (0.95-1.19) | 0.93 (0.83-1.05) | 0.46 | 1.00 (0.84-1.19) | 1.03 (0.87-1.22) | 0.88 (0.56-1.37) |
| Thyroid gland |  |  |  |  |  |  |  |  |
| *5-year mortality* | 848 | 90 | 1.02 (0.61-1.68) | 0.94 (0.55-1.61) | 0.71 | 1.16 (0.54-2.48) | 1.22 (0.62-2.41) | 3.04 (1.22-7.61) |
| *10-year mortality* |  | 104 | 0.79 (0.49-1.25) | 0.75 (0.46-1.24) | 0.78 | 1.01 (0.49-2.06) | 1.20 (0.63-2.28) | 2.66 (1.07-6.61) |
| *15-year mortality* |  | 116 | 0.80 (0.52-1.24) | 0.71 (0.44-1.14) | 0.45 | 0.97 (0.48-1.97) | 1.30 (0.72-2.35) | 2.89 (1.25-6.67) |
| GASTROINTESTINAL CANCER | | |  |  |  |  |  |  |
| Esophagus |  |  |  |  |  |  |  |  |
| *5-year mortality* | 991 | 700 | 0.88 (0.74-1.05) | 0.93 (0.76-1.13) | 0.48 | 1.00 (0.75-1.32) | 1.09 (0.87-1.37) | 1.15 (0.75-1.76) |
| *10-year mortality* |  | 719 | 0.86 (0.73-1.31) | 0.92 (0.76-1.11) | 0.42 | 1.00 (0.76-1.31) | 1.10 (0.88-1.37) | 1.12 (0.73-1.72) |
| *15-year mortality* |  | 721 | 0.86 (0.73-1.03) | 0.92 (0.76-1.11) | 0.40 | 1.01 (0.77-1.32) | 1.09 (0.87-1.37) | 1.12 (0.73-1.71) |
| Stomach |  |  |  |  |  |  |  |  |
| *5-year mortality* | 1,269 | 791 | 0.98 (0.83-1.16) | 0.93 (0.78-1.12) | 0.40 | 0.95 (0.74-1.23) | 1.06 (0.85-1.32) | 1.23 (0.79-1.91) |
| *10-year mortality* |  | 815 | 0.97 (0.82-1.14) | 0.91 (0.76-1.09) | 0.24 | 0.99 (0.77-1.27) | 1.07 (0.86-1.32) | 1.27 (0.83-1.95) |
| *15-year mortality* |  | 822 | 0.97 (0.82-1.14) | 0.91 (0.76-1.09) | 0.25 | 0.98 (0.76-1.25) | 1.05 (0.85-1.31) | 1.26 (0.82-1.93) |
| Pancreas |  |  |  |  |  |  |  |  |
| *5-year mortality* | 1,809 | 1,281 | 0.92 (0.81-1.05) | 0.83 (0.72-0.96) | 0.048 | 1.07 (0.89-1.29) | 0.96 (0.80-1.15) | 1.40 (0.90-2.18) |
| *10-year mortality* |  | 1,305 | 0.92 (0.80-1.04) | 0.81 (0.70-0.94) | 0.025 | 1.08 (0.89-1.30) | 0.97 (0.81-1.16) | 1.39 (0.89-2.17) |
| *15-year mortality* |  | 1,311 | 0.91 (0.80-1.04) | 0.81 (0.70-0.94) | 0.023 | 1.07 (0.89-1.29) | 0.97 (0.81-1.16) | 1.39 (0.89-2.16) |
| Liver, bile ducts and gallbladder |  |  |  |  |  |  |  |  |
| *5-year mortality* | 1,573 | 1,062 | 0.85 (0.74-0.98) | 0.87 (0.74-1.03) | 0.031 | 1.09 (0.89-1.34) | 1.00 (0.82-1.22) | 1.17 (0.78-1.76) |
| *10-year mortality* |  | 1,084 | 0.87 (0.75-1.00) | 0.87 (0.74-1.02) | 0.036 | 1.07 (0.87-1.32) | 1.02 (0.83-1.24) | 1.19 (0.80-1.78) |
| *15-year mortality* |  | 1,087 | 0.87 (0.76-1.00) | 0.87 (0.73-1.02) | 0.037 | 1.08 (0.88-1.33) | 1.02 (0.83-1.24) | 1.19 (0.80-1.79) |
| Colon |  |  |  |  |  |  |  |  |
| *5-year mortality* | 4,265 | 1,314 | 1.01 (0.89-1.14) | 0.95 (0.82-1.10) | 0.33 | 1.14 (0.94-1.37) | 0.99 (0.81-1.19) | 1.13 (0.78-1.62) |
| *10-year mortality* |  | 1,414 | 1.00 (0.88-1.13) | 0.92 (0.80-1.05) | 0.11 | 1.11 (0.93-1.33) | 0.97 (0.81-1.17) | 1.09 (0.76-1.55) |
| *15-year mortality* |  | 1,443 | 1.00 (0.88-1.12) | 0.91 (0.79-1.05) | 0.088 | 1.10 (0.92-1.32) | 0.97 (0.81-1.17) | 1.10 (0.78-1.57) |
| Rectum |  |  |  |  |  |  |  |  |
| *5-year mortality* | 3,123 | 829 | 0.94 (0.80-1.11) | 0.82 (0.68-0.98) | 0.021 | 1.00 (0.79-1.28) | 1.47 (1.17-1.85) | 1.48 (0.91-2.40) |
| *10-year mortality* |  | 929 | 0.96 (0.82-1.11) | 0.82 (0.69-0.98) | 0.015 | 0.95 (0.75-1.20) | 1.42 (1.14-1.77) | 1.33 (0.82-2.15) |
| *15-year mortality* |  | 950 | 0.96 (0.83-1.12) | 0.83 (0.70-0.99) | 0.021 | 0.97 (0.77-1.21) | 1.41 (1.13-1.75) | 1.30 (0.80-2.10) |
| UROLOGICAL CANCER | | |  |  |  |  |  |  |
| Kidney |  |  |  |  |  |  |  |  |
| *5-year mortality* | 2,288 | 542 | 1.09 (0.90-1.34) | 1.07 (0.86-1.33) | 0.51 | 0.69 (0.48-1.01) | 1.36 (1.07-1.73) | 1.82 (1.20-2.78) |
| *10-year mortality* |  | 605 | 1.07 (0.89-1.30) | 1.01 (0.82-1.25) | 0.86 | 0.68 (0.47-0.97) | 1.45 (1.16-1.81) | 1.84 (1.24-2.74) |
| *15-year mortality* |  | 621 | 1.05 (0.87-1.26) | 0.99 (0.80-1.21) | 0.98 | 0.69 (0.49-0.98) | 1.45 (1.16-1.81) | 1.88 (1.27-2.77) |
| Bladder |  |  |  |  |  |  |  |  |
| *5-year mortality* | 3,078 | 383 | 0.90 (0.71-1.14) | 0.72 (0.55-0.94) | 0.022 | 0.75 (0.51-1.10) | 0.94 (0.64-1.40) | 2.10 (1.11-3.96) |
| *10-year mortality* |  | 447 | 0.91 (0.73-1.13) | 0.76 (0.60-0.98) | 0.034 | 0.88 (0.63-1.23) | 1.03 (0.73-1.47) | 2.25 (1.26-4.02) |
| *15-year mortality* |  | 461 | 0.92 (0.74-1.15) | 0.78 (0.62-1.00) | 0.047 | 0.90 (0.65-1.25) | 1.02 (0.72-1.45) | 2.20 (1.23-3.93) |
| Prostate |  |  |  |  |  |  |  |  |
| *5-year mortality* | 19,686 | 948 | 0.95 (0.82-1.11) | 0.83 (0.70-0.98) | 0.050 | 0.84 (0.66-1.06) | 1.30 (1.02-1.65) | 2.44 (1.41-4.23) |
| *10-year mortality* |  | 1,194 | 0.94 (0.82-1.07) | 0.84 (0.72-0.97) | 0.036 | 0.85 (0.69-1.04) | 1.23 (0.98-1.53) | 2.51 (1.53-4.13) |
| *15-year mortality* |  | 1,235 | 0.94 (0.82-1.07) | 0.82 (0.71-0.95) | 0.013 | 0.85 (0.70-1.04) | 1.27 (1.02-1.57) | 2.63 (1.62-4.25) |
| HEMATOLOGICAL CANCER | | |  |  |  |  |  |  |
| Leukemia |  |  |  |  |  |  |  |  |
| *5-year mortality* | 2,698 | 639 | 1.05 (0.86-1.27) | 1.00 (0.81-1.23) | 0.86 | 1.39 (1.07-1.81) | 0.95 (0.72-1.26) | 1.31 (0.75-2.27) |
| *10-year mortality* |  | 730 | 1.05 (0.88-1.26) | 0.96 (0.79-1.16) | 0.74 | 1.39 (1.08-1.78) | 1.00 (0.78-1.29) | 1.35 (0.81-2.26) |
| *15-year mortality* |  | 768 | 1.00 (0.84-1.20) | 0.97 (0.80-1.16) | 0.86 | 1.42 (1.12-1.82) | 1.05 (0.82-1.34) | 1.32 (0.79-2.20) |
| Myeloma |  |  |  |  |  |  |  |  |
| *5-year mortality* | 1,209 | 251 | 1.14 (0.83-1.57) | 1.20 (0.86-1.66) | 0.23 | 1.65 (1.06-2.58) | 1.40 (0.94-2.10) | 0.71 (0.17-2.87) |
| *10-year mortality* |  | 332 | 1.15 (0.87-1.52) | 1.18 (0.89-1.57) | 0.18 | 1.24 (0.81-1.89) | 1.22 (0.85-1.77) | 0.49 (0.12-1.98) |
| *15-year mortality* |  | 358 | 1.21 (0.93-1.58) | 1.24 (0.94-1.64) | 0.070 | 1.09 (0.71-1.67) | 1.23 (0.87-1.75) | 0.66 (0.21-2.06) |
| Hodgkin lymphoma |  |  |  |  |  |  |  |  |
| *5-year mortality* | 1,112 | 107 | 0.93 (0.59-1.47) | 0.81 (0.49-1.35) | 0.67 | 1.55 (0.85-2.84) | 0.89 (0.47-1.68) | 2.40 (0.95-6.07) |
| *10-year mortality* |  | 141 | 0.98 (0.66-1.46) | 0.77 (0.49-1.20) | 0.41 | 1.22 (0.69-2.17) | 0.94 (0.56-1.61) | 2.02 (0.87-4.68) |
| *15-year mortality* |  | 160 | 0.97 (0.67-1.40) | 0.72 (0.47-1.09) | 0.26 | 1.10 (0.64-1.91) | 0.98 (0.60-1.60) | 1.74 (0.75-4.01) |
| Non-Hodgkin lymphoma |  |  |  |  |  |  |  |  |
| *5-year mortality* | 3,261 | 530 | 0.85 (0.69-1.04) | 0.78 (0.63-0.97) | 0.011 | 0.82 (0.58-1.16) | 1.14 (0.85-1.51) | 1.28 (0.72-2.28) |
| *10-year mortality* |  | 616 | 0.86 (0.71-1.04) | 0.79 (0.64-0.97) | 0.007 | 0.83 (0.61-1.14) | 1.17 (0.90-1.52) | 1.44 (0.86-2.41) |
| *15-year mortality* |  | 655 | 0.86 (0.71-1.03) | 0.77 (0.63-0.94) | 0.004 | 0.83 (0.61-1.12) | 1.17 (0.91-1.51) | 1.56 (0.96-2.54) |

Hazard ratios for mortality in each site-specific cancer by cardiorespiratory fitness and body composition. Analyses adjusted for year of conscription, conscription center, age at conscription, and date of cancer diagnosis. ^a^Evaluated with maximal aerobic workload and transformed to a standardized score (1-9) and categorized into low CRF (1-5), moderate CRF (6-7), and high CRF (8-9). ^b^Analyzed with the 9-grade CRF scale. CRF = Cardiorespiratory fitness. BMI = Body mass index. Categorized into underweight (<18.5 kg/m2), normal weight (18.5-24.9 kg/m2), overweight (25-29.9 kg/m2), and obesity (≥30 kg/m2). Reference is normal weight.

Supplementary table S5. Five-year mortality per cancer site, with and without adjusting for parental education

|  |  | Cardiorespiratory fitness (ref = low) | | BMI (ref = Normal weight) | | |
| --- | --- | --- | --- | --- | --- | --- |
|  |  | **Moderate** | **High** | **Underweight** | **Overweight** | **Obesity** |
|  | **N cases** | **HRR (95% CI)** | **HRR (95% CI)** | **HRR (95% CI)** | **HRR (95% CI)** | **HRR (95% CI)** |
| Any cancer site | 65,118 | 0.83 (0.79-0.87) | 0.69 (0.65-0.73) | 0.98 (0.90-1.05) | 1.39 (1.29-1.50) | 1.94 (1.65-2.28) |
| *Adjusted* |  | 0.84 (0.79-0.88) | 0.70 (0.66-0.74) | 0.99 (0.91-1.06) | 1.37 (1.27-1.47) | 1.89 (1.61-2.22) |
| Malignant skin | 23,136 | 0.81 (0.68-0.97) | 0.79 (0.65-0.94) | 0.80 (0.60-1.06) | 1.50 (1.17-1.91) | 1.64 (0.82-3.30) |
| *Adjusted* |  | 0.82 (0.69-0.98) | 0.81 (0.67-0.97) | 0.80 (0.60-1.06) | 1.47 (1.15-1.88) | 1.61 (0.80-3.23) |
| Bronchi and lung | 1,594 | 0.76 (0.65-0.88) | 0.78 (0.66-0.92) | 0.94 (0.77-1.14) | 1.11 (0.87-1.41) | 1.16 (0.73-1.84) |
| *Adjusted* |  | 0.76 (0.65-0.88) | 0.78 (0.66-0.93) | 0.94 (0.77-1.14) | 1.10 (0.86-1.40) | 1.17 (0.74-1.85) |
| Head and neck | 2,747 | 0.82 (0.66-1.01) | 0.64 (0.51-0.82) | 0.92 (0.67-1.25) | 1.39 (1.02-1.88) | 1.23 (0.55-2.77) |
| *Adjusted* |  | 0.83 (0.67-1.03) | 0.66 (0.52-0.84) | 0.92 (0.67-1.27) | 1.37 (1.01-1.86) | 1.23 (0.55-2.77) |
| Central nervous system | 2,078 | 1.00 (0.85-1.16) | 0.88 (0.75-1.04) | 1.00 (0.79-1.28) | 0.91 (0.72-1.16) | 0.81 (0.43-1.51) |
| *Adjusted* |  | 1.01 (0.86-1.17) | 0.89 (0.75-1.05) | 1.01 (0.79-1.29) | 0.90 (0.71-1.14) | 0.79 (0.42-1.49) |
| Thyroid gland | 709 | 1.04 (0.57-1.89) | 0.75 (0.38-1.46) | 0.46 (0.11-1.93) | 1.30 (0.55-3.08) | 1.47 (0.35-6.11) |
| *Adjusted* |  | 1.01 (0.55-1.85) | 0.72 (0.36-1.41) | 0.48 (0.11-2.02) | 1.39 (0.59-3.32) | 1.49 (0.36-6.21) |
| GASTROINTESTINAL CANCER | | |  |  |  |  |
| Esophagus | 676 | 0.88 (0.71-1.10) | 0.90 (0.71-1.15) | 1.08 (0.76-1.55) | 1.22 (0.93-1.59) | 2.11 (1.27-3.51) |
| *Adjusted* |  | 0.88 (0.71-1.09) | 0.90 (0.70-1.14) | 1.07 (0.75-1.54) | 1.22 (0.93-1.60) | 2.12 (1.27-3.53) |
| Stomach | 885 | 0.93 (0.76-1.15) | 1.01 (0.81-1.26) | 1.09 (0.78-1.50) | 1.08 (0.83-1.39) | 2.03 (1.27-3.24) |
| *Adjusted* |  | 0.93 (0.75-1.14) | 0.99 (0.79-1.24) | 1.10 (0.80-1.52) | 1.10 (0.85-1.42) | 1.96 (1.23-3.13) |
| Pancreas | 1,252 | 0.89 (0.75-1.04) | 0.86 (0.72-1.03) | 1.09 (0.86-1.38) | 0.93 (0.74-1.16) | 1.28 (0.66-2.48) |
| *Adjusted* |  | 0.88 (0.75-1.04) | 0.87 (0.73-1.04) | 1.08 (0.85-1.37) | 0.91 (0.73-1.14) | 1.24 (0.64-2.41) |
| Liver, bile ducts and gallbladder | 1,096 | 0.84 (0.71-1.00) | 0.88 (0.72-1.08) | 1.31 (1.01 (1.68) | 1.00 (0.78-1.29) | 1.25 (0.75-2.08) |
| *Adjusted* |  | 0.84 (0.70-1.00) | 0.87 (0.71-1.07) | 1.31 (1.01-1.69) | 1.01 (0.79-1.29) | 1.24 (0.74-2.06) |
| Colon | 3,202 | 0.99 (0.84-1.15) | 0.99 (0.83-1.17) | 1.11 (0.88-1.40) | 0.95 (0.75-1.20) | 1.31 (0.85-2.03) |
| *Adjusted* |  | 0.99 (0.85-1.16) | 1.00 (0.84-1.19) | 1.12 (0.89-1.42) | 0.95 (0.75-1.20) | 1.29 (0.84-2.00) |
| Rectum | 2,330 | 0.96 (0.79-1.17) | 0.82 (0.66-1.03) | 0.95 (0.70-1.29) | 1.50 (1.14-1.97) | 1.77 (1.04-3.03) |
| *Adjusted* |  | 0.96 (0.79-1.17) | 0.83 (0.66-1.03) | 0.96 (0.70-1.31) | 1.48 (1.13-1.95) | 1.71 (1.00-2.94) |
| UROLOGICAL CANCER | | |  |  |  |  |
| Kidney | 1,748 | 1.15 (0.90-1.46) | 1.03 (0.79-1.35) | 0.57 (0.35-0.93) | 1.29 (0.96-1.74) | 1.43 (0.78-2.62) |
| *Adjusted* |  | 1.15 (0.90-1.47) | 1.03 (0.79-1.35) | 0.58 (0.35-0.94) | 1.29 (0.96-1.74) | 1.42 (0.77-2.61) |
| Bladder | 2,269 | 0.76 (0.56-1.03) | 0.71 (0.51-0.98) | 0.72 (0.44-1.17) | 0.75 (0.43-1.33) | 2.26 (0.99-5.18) |
| *Adjusted* |  | 0.77 (0.57-1.05) | 0.72 (0.52-1.00) | 0.73 (0.45-1.19) | 0.73 (0.42-1.29) | 2.23 (0.97-5.10) |
| Prostate | 14,240 | 0.93 (0.77-1.13) | 0.88 (0.71-1.07) | 0.77 (0.57-1.04) | 1.25 (0.92-1.69) | 2.27 (1.07-4.78) |
| *Adjusted* |  | 0.93 (0.77-1.13) | 0.89 (0.72-1.09) | 0.77 (0.57-1.05) | 1.23 (0.91-1.67) | 2.23 (1.06-4.72) |
| HEMATOLOGICAL CANCER | | |  |  |  |  |
| Leukemia | 2,022 | 1.09 (0.84-1.40) | 0.98 (0.75-1.30) | 1.73 (1.22-2.45) | 1.13 (0.81-1.59) | 0.73 (0.27-1.95) |
| *Adjusted* |  | 1.10 (0.85-1.42) | 1.01 (0.76-1.34) | 1.72 (1.22-2.44) | 1.12 (0.80-1.57) | 0.70 (0.26-1.88) |
| Myeloma | 919 | 1.23 (0.83-1.82) | 1.09 (0.72-1.67) | 1.89 (1.10-3.25) | 1.63 (0.99-2.68) | 0.45 (0.06-3.23) |
| *Adjusted* |  | 1.23 (0.83-1.82) | 1.14 (0.75-1.75) | 1.94 (1.13-3.33) | 1.58 (0.96-2.60) | 0.41 (0.06-2.95) |
| Hodgkin lymphoma | 907 | 0.67 (0.34-1.32) | 0.73 (0.35-1.50) | 2.63 (1.23-5.60) | 0.71 (0.25-2.02) | 2.52 (0.58-10.99) |
| *Adjusted* |  | 0.67 (0.34-1.31) | 0.71 (0.34-1.47) | 2.67 (1.25-5.71) | 0.70 (0.25-2.00) | 2.51 (0.58-10.9) |
| Non-Hodgkin lymphoma | 2,573 | 0.79 (0.61-1.02) | 0.72 (0.54-0.95) | 0.73 (0.46-1.17) | 1.10 (0.76-1.58) | 1.08 (0.51-2.30) |
| *Adjusted* |  | 0.79 (0.61-1.02) | 0.73 (0.55-0.96) | 0.73 (0.46-1.17) | 1.09 (0.76-1.57) | 1.08 (0.51-2.29) |

Hazard ratios for mortality in each site-specific cancer according to cardiorespiratory fitness and BMI. Analyses adjusted for year of conscription, conscription site, age at conscription, and parental level of education. BMI = Body mass index. Categorized into underweight (<18.5 kg/m2), normal weight (18.5-24.9 kg/m2), overweight (25-29.9 kg/m2), and obesity (≥30 kg/m2). Reference is normal weight.

Supplementary table S6. Hazard ratios for five-year mortality after site-specific cancers, adjusted for muscle strength at conscription.

|  |  |  | Cardiorespiratory fitness (ref = low) | | | BMI (ref = Normal weight) | | |
| --- | --- | --- | --- | --- | --- | --- | --- | --- |
| Cancer site | **n cases** | **n (%) deaths** | **Moderate** | **High** | **p-value for linear trend^a^** | **Underweight** | **Overweight** | **Obesity** |
|  |  |  | **HRR (95% CI)** | **HRR (95% CI)** |  | **HRR (95% CI)** | **HRR (95% CI)** | **HRR (95% CI)** |
| Any cancer site | 82,302 | 13,133 | 0.85 (0.82-0.89) | 0.71 (0.68-0.74) | <0.001 | 0.94 (0.89-1.00) | 1.40 (1.32-1.48) | 1.90 (1.67-2.15) |
| Malignant skin | 27,597 | 1,169 | 0.82 (0.68-0.98) | 0.74 (0.60-0.92) | 0.008 | 0.91 (0.74-1.13) | 1.62 (1.33-1.98) | 2.33 (1.44-3.78) |
| Bronchi and lung | 2,443 | 1,662 | 0.83 (0.74-0.93) | 0.81 (0.70-0.93) | 0.001 | 0.91 (0.78-1.06) | 1.04 (0.86-1.27) | 1.18 (0.81-1.70) |
| Head and neck | 3,459 | 697 | 0.84 (0.71-1.01) | 0.71 (0.58-0.87) | 0.001 | 1.06 (0.82-1.36) | 1.51 (1.18-1.94) | 1.36 (0.70-2.65) |
| Central nervous system | 2,841 | 1,537 | 1.04 (0.92-1.17) | 0.88 (0.77-1.01) | 0.18 | 1.06 (0.87-1.28) | 1.03 (0.85-1.25) | 0.94 (0.58-1.53) |
| Thyroid gland | 803 | 89 | 0.99 (0.59-1.64) | 0.95 (0.55-1.63) | 0.62 | 1.27 (0.59-2.74) | 1.48 (0.75-2.94) | 4.50 (1.78-11.36) |
| GASTROINTESTINAL CANCER | | |  |  |  |  |  |  |
| Esophagus | 968 | 680 | 0.88 (0.73-1.05) | 0.92 (0.75-1.12) | 0.42 | 1.01 (0.76-1.36) | 1.11 (0.88-1.40) | 1.13 (0.73-1.74) |
| Stomach | 1,237 | 770 | 0.98 (0.82-1.16) | 0.93 (0.77-1.13) | 0.55 | 0.92 (0.70-1.20) | 1.06 (0.85-1.33) | 1.19 (0.76-1.88) |
| Pancreas | 1,774 | 1,258 | 0.95 (0.83-1.09) | 0.87 (0.75-1.01) | 0.18 | 1.05 (0.86-1.28) | 0.95 (0.78-1.14) | 1.40 (0.90-2.19) |
| Liver, bile ducts and gallbladder | 1,549 | 1,046 | 0.86 (0.74-0.99) | 0.87 (0.73-1.03) | 0.05 | 1.08 (0.87-1.35) | 1.02 (0.83-1.25) | 1.08 (0.70-1.67) |
| Colon | 4,165 | 1,285 | 0.99 (0.87-1.13) | 0.93 (0.80-1.08) | 0.19 | 1.10 (0.90-1.34) | 0.97 (0.80-1.18) | 1.08 (0.75-1.56) |
| Rectum | 3,043 | 810 | 0.96 (0.82-1.13) | 0.85 (0.71-1.03) | 0.05 | 0.97 (0.75-1.24) | 1.50 (1.19-1.90) | 1.51 (0.93-2.47) |
| UROLOGICAL CANCER | | |  |  |  |  |  |  |
| Kidney | 2,247 | 526 | 1.12 (0.91-1.37) | 1.11 (0.88-1.40) | 0.34 | 0.68 (0.47-1.00) | 1.37 (1.06-1.76) | 1.90 (1.23-2.96) |
| Bladder | 3,017 | 378 | 0.95 (0.74-1.21) | 0.76 (0.58-1.00) | 0.10 | 0.72 (0.49-1.06) | 0.99 (0.66-1.48) | 2.16 (1.14-4.10) |
| Prostate | 19,355 | 927 | 0.95 (0.82-1.12) | 0.83 (0.70-0.99) | 0.10 | 0.82 (0.65-1.04) | 1.32 (1.03-1.69) | 2.33 (1.31-4.13) |
| HEMATOLOGICAL CANCER | | |  |  |  |  |  |  |
| Leukemia | 2,600 | 626 | 1.02 (0.84-1.24) | 1.02 (0.82-1.25) | 0.68 | 1.37 (1.04-1.81) | 0.99 (0.75-1.32) | 1.40 (0.80-2.44) |
| Myeloma | 1,177 | 246 | 1.13 (0.82-1.56) | 1.20 (0.85-1.68) | 0.23 | 1.74 (1.10-2.75) | 1.47 (0.97-2.23) | 0.72 (0.18-2.93) |
| Hodgkin lymphoma | 1,054 | 104 | 0.89 (0.56-1.42) | 0.77 (0.46-1.30) | 0.65 | 1.60 (0.85-3.00) | 0.82 (0.41-1.61) | 2.57 (1.00-6.57) |
| Non-Hodgkin lymphoma | 3,157 | 517 | 0.80 (0.65-0.98) | 0.73 (0.58-0.92) | 0.002 | 0.81 (0.57-1.16) | 1.08 (0.80-1.46) | 1.18 (0.66-2.11) |

Analyses adjusted for site of conscription, year of conscription, age at conscription. BMI = Body mass index. Categorized into underweight (<18.5 kg/m2), normal weight (18.5-24.9 kg/m2), overweight (25-29.9 kg/m2), and obesity (≥30 kg/m2). Reference is normal weight.

Supplementary table S7. Associations between cardiorespiratory fitness in youth and five-year mortality per cancer site for all cancer cases, regardless of previous cancers. Stratified by year of conscription.

|  | Conscription 1968-1979 | | | Conscription 1980-2005 | | |
| --- | --- | --- | --- | --- | --- | --- |
| Cancer site | **N cases** | **Moderate** | **High** | **N cases** | **Moderate** | **High** |
|  |  | **HR (95% CI)** | **HR (95% CI)** |  | **HR (95% CI)** | **HR (95% CI)** |
| Any cancer site | 56,360 | 0.83 (0.79-0.87) | 0.68 (0.65-0.72) | 28,261 | 0.90 (0.83-0.98) | 0.73 (0.66-0.80) |
| Malignant skin | 17,367 | 0.83 (0.71-0.99) | 0.82 (0.69-0.97) | 10,992 | 0.98 (0.76-1.28) | 0.74 (0.55-0.99) |
| Bronchi and lung | 2,049 | 0.87 (0.77-0.99) | 0.83 (0.72-0.95) | 453 | 0.69 (0.51-0.92) | 0.84 (0.58-1.20) |
| Head and neck | 2,418 | 0.82 (0.68-1.00) | 0.72 (0.58-0.89) | 1,131 | 0.76 (0.52-1.10) | 0.50 (0.30-0.82) |
| Central nervous system | 1,570 | 1.03 (0.88-1.21) | 0.83 (0.70-0.98) | 1,367 | 1.11 (0.91-1.35) | 1.02 (0.81-1.28) |
| Thyroid gland | 403 | 0.84 (0.46-1.55) | 0.80 (0.43-1.49) | 445 | 1.58 (0.57-4.36) | 1.23 (0.36-4.20) |
| GASTROINTESTINAL CANCER | | |  |  |  |  |
| Esophagus | 781 | 0.86 (0.70-1.04) | 0.94 (0.76-1.17) | 210 | 1.09 (0.73-1.64) | 0.89 (0.53-1.50) |
| Stomach | 934 | 0.92 (0.76-1.12) | 0.87 (0.70-1.08) | 335 | 1.27 (0.89-1.80) | 1.19 (0.81-1.73) |
| Pancreas | 1,401 | 0.93 (0.80-1.08) | 0.80 (0.68-0.94) | 408 | 0.79 (0.58-1.07) | 0.88 (0.61-1.28) |
| Liver, bile ducts and gallbladder | 1,183 | 0.83 (0.71-0.98) | 0.84 (0.70-1.02) | 390 | 0.85 (0.63-1.15) | 0.93 (0.65-1.33) |
| Colon | 2,922 | 0.92 (0.79-1.07) | 0.87 (0.73-1.03) | 1,343 | 1.27 (1.00-1.61) | 1.17 (0.88-1.55) |
| Rectum | 2,242 | 0.97 (0.80-1.17) | 0.84 (0.68-1.03) | 881 | 0.87 (0.63-1.20) | 0.80 (0.54-1.18) |
| UROLOGICAL CANCER | | |  |  |  |  |
| Kidney | 1,586 | 1.06 (0.84-1.34) | 1.05 (0.82-1.34) | 702 | 1.14 (0.76-1.71) | 0.94 (0.55-1.62) |
| Bladder | 2,370 | 0.95-0.73-1.23) | 0.70 (0.52-0.94) | 708 | 0.79 (0.42-1.51) | 0.90 (0.46-1.79) |
| Prostate | 16,937 | 0.96 (0.82-1.13) | 0.82 (0.69-0.98) | 2,749 | 0.72 (0.44-1.19) | 0.72 (0.42-1.26) |
| HEMATOLOGICAL CANCER | | |  |  |  |  |
| Leukemia | 1,688 | 0.94 (0.74-1.19) | 0.98 (0.77-1.25) | 1,010 | 1.16 (0.84-1.62) | 0.91 (0.60-1.37) |
| Myeloma | 858 | 1.16 (0.80-1.68) | 1.38 (0.95-2.00) | 351 | 0.92 (0.50-1.70) | 0.50 (0.21-1.17) |
| Hodgkin lymphoma | 449 | 1.36 (0.74-2.50) | 0.68 (0.33-1.41) | 663 | 0.55 (0.26-1.14) | 0.88 (0.42-1.82) |
| Non-Hodgkin lymphoma | 1,878 | 0.85 (0.66-1.09) | 0.74 (0.56-0.96) | 1,383 | 0.77 (0.53-1.12) | 0.85 (0.56-1.28) |

Analyses adjusted for year of conscription, conscription center, age at conscription, body mass index at conscription, and date of cancer diagnosis. Cardiorespiratory fitness evaluated with maximal aerobic workload and transformed to a standardized score (1-9) and categorized into low CRF (1-5), moderate CRF (6-7), and high CRF (8-9), with low being the reference in the analyses.

Supplementary table S8. Associations between body mass index in youth and five-year mortality per cancer site for all cancer cases, regardless of previous cancers. Stratified by year of conscription.

|  | Conscription 1968-1979 | | | Conscription 1980-2005 | | |
| --- | --- | --- | --- | --- | --- | --- |
| Cancer site | **N cases** | **Overweight** | **Obesity** | **N cases** | **Overweight** | **Obesity** |
|  |  | **HR (95% CI)** | **HR (95% CI)** |  | **HR (95% CI)** | **HR (95% CI)** |
| Any cancer site | 56,360 | 1.42 (1.33-1.52) | 1.89 (1.62-2.21) | 28,261 | 1.26 (1.13-1.41) | 1.92 (1.56-2.36) |
| Malignant skin | 17,367 | 1.61 (1.28-2.03) | 2.15 (1.22-3.82) | 10,992 | 1.25 (0.87-1.79) | 1.71 (0.70-4.18) |
| Bronchi and lung | 2,049 | 0.98 (0.78-1.21) | 1.06 (0.68-1.65) | 453 | 1.31 (0.88-1.98) | 1.36 (0.70-2.67) |
| Head and neck | 2,418 | 1.54 (1.16-2.05) | 1.38 (0.61-3.09) | 1,131 | 1.40 (0.88-2.23) | 1.69 (0.62-4.62) |
| Central nervous system | 1,570 | 1.06 (0.83-1.36) | 1.13 (0.62-2.05) | 1,367 | 1.02 (0.77-1.35) | 0.70 (0.31-1.59) |
| Thyroid gland | 403 | 1.43 (0.67-3.03) | 3.47 (1.21-9.97) | 445 | 0.52 (0.07-4.00) | 1.85 (0.24-14.23) |
| GASTROINTESTINAL CANCER | | |  |  |  |  |
| Esophagus | 781 | 1.07 (0.83-1.38) | 0.78 (0.40-1.51) | 210 | 1.21 (0.70-2.09) | 1.89 (1.01-3.53) |
| Stomach | 934 | 1.12 (0.87-1.46) | 0.73 (0.38-1.42) | 335 | 1.05 (0.69-1.61) | 2.95 (1.57-5.53) |
| Pancreas | 1,401 | 0.99 (0.80-1.21) | 1.55 (0.94-2.55) | 408 | 0.80 (0.53-1.23) | 0.92 (0.34-2.51) |
| Liver, bile ducts and gallbladder | 1,183 | 1.05 (0.83-1.33) | 1.31 (0.81-2.13) | 390 | 0.84 (0.56-1.26) | 0.91 (0.41-2.02) |
| Colon | 2,922 | 0.96 (0.76-1.22) | 1.19 (0.75-1.91) | 1,343 | 1.07 (0.77-1.47) | 1.17 (0.66-2.10) |
| Rectum | 2,242 | 1.40 (1.06-1.85) | 1.36 (0.73-2.56) | 881 | 1.57 (1.04-2.38) | 1.74 (0.81-3.73) |
| UROLOGICAL CANCER | | |  |  |  |  |
| Kidney | 1,586 | 1.41 (1.07-1.86) | 1.86 (1.14-3.03) | 702 | 1.26 (0.76-2.09) | 1.58 (0.68-3.66) |
| Bladder | 2,370 | 0.84 (0.52-1.33) | 2.08 (1.02-4.24) | 708 | 1.26 (0.58-2.72) | 1.85 (0.43-8.00) |
| Prostate | 16,937 | 1.43 (1.12-1.83) | 2.57 (1.41-4.66) | 2,749 | 0.38 (0.12-1.20) | 1.94 (0.47-8.03) |
| HEMATOLOGICAL CANCER | | |  |  |  |  |
| Leukemia | 1,688 | 1.08 (0.77-1.53) | 1.17 (0.55-2.49) | 1,010 | 0.81 (0.49-1.32) | 1.35 (0.59-3.09) |
| Myeloma | 858 | 1.46 (0.93-2.29) | 1.07 (0.26-4.37) | 351 | 0.99 (0.39-2.51) | NA |
| Hodgkin lymphoma | 449 | 0.96 (0.41-2.26) | 1.34 (0.32-5.65) | 663 | 0.81 (0.31-2.11) | 3.90 (1.09-14.00) |
| Non-Hodgkin lymphoma | 1,878 | 1.28 (0.90-1.82) | 1.60 (0.82-3.11) | 1,383 | 0.91 (0.56-1.49) | 0.81 (0.26-2.59) |

Analyses adjusted for year of conscription, conscription center, age at conscription, cardiorespiratory fitness at conscription, and date of cancer diagnosis. BMI = Body mass index. Categorized into underweight (<18.5 kg/m2), normal weight (18.5-24.9 kg/m2), overweight (25-29.9 kg/m2), and obesity (≥30 kg/m2). Reference is normal weight.

Supplementary table S9. Associations between cardiorespiratory fitness in youth and five-year mortality per cancer site for all cancer cases, regardless of previous cancers. Stratified by BMI at conscription.

|  | BMI <25 | | | BMI ≥25 | | |
| --- | --- | --- | --- | --- | --- | --- |
| Cancer site | **N cases** | **Moderate** | **High** | **N cases** | **Moderate** | **High** |
|  |  | **HR (95% CI)** | **HR (95% CI)** |  | **HR (95% CI)** | **HR (95% CI)** |
| Any cancer site | 76,971 | 0.85 (0.82-0.89) | 0.70 (0.67-0.73) | 7,650 | 0.82 (0.72-0.92) | 0.69 (0.61-0.79) |
| Malignant skin | 26,170 | 0.88 (0.76-1.01) | 0.80 (0.69-0.93) | 2,189 | 0.92 (0.59-1.44) | 0.88 (0.56-1.39) |
| Bronchi and lung | 2,283 | 0.86 (0.76-0.96) | 0.87 (0.76-0.99) | 219 | 0.69 (0.46-1.03) | 0.60 (0.38-0.93) |
| Head and neck | 3,202 | 0.88 (0.73-1.05) | 0.69 (0.56-0.85) | 347 | 0.51 (0.29-0.90) | 0.62 (0.37-1.06) |
| Central nervous system | 2,651 | 1.04 (0.92-1.17) | 0.89 (0.78-1.01) | 286 | 1.10 (0.73-1.68) | 0.83 (0.55-1.26) |
| Thyroid gland | 748 | 0.99 (0.58-1.68) | 0.85 (0.47-1.52) | 100 | 0.92 (0.19-4.37) | 1.03 (0.22-4.96) |
| GASTROINTESTINAL CANCER | | |  |  |  |  |
| Esophagus | 841 | 0.89 (0.74-1.08) | 0.96 (0.78-1.19) | 150 | 0.88 (0.54-1.43) | 0.91 (0.55-1.50) |
| Stomach | 1,086 | 0.95 (0.80-1.14) | 1.00 (0.82-1.21) | 183 | 1.38 (0.84-2.26) | 0.92 (0.54-1.59) |
| Pancreas | 1,593 | 0.90 (0.79-1.04) | 0.84 (0.72-0.97) | 216 | 0.89 (0.58-1.38) | 0.66 (0.42-1.04) |
| Liver, bile ducts and gallbladder | 1,376 | 0.87 (0.75-1.00) | 0.83 (0.69-0.99) | 197 | 0.60 (0.39-0.94) | 0.91 (0.59-1.38) |
| Colon | 3,776 | 1.00 (0.88-1.14) | 0.93 (0.80-1.08) | 489 | 0.90 (0.61-1.34) | 0.88 (0.58-1.36) |
| Rectum | 2,818 | 0.93 (0.78-1.10) | 0.84 (0.70-1.02) | 305 | 1.02 (0.61-1.70) | 0.66 (0.38-1.13) |
| UROLOGICAL CANCER | | |  |  |  |  |
| Kidney | 1,949 | 1.16 (0.93-1.44) | 1.14 (0.90-1.46) | 339 | 0.86 (0.53-1.41) | 0.82 (0.49-1.38) |
| Bladder | 2,793 | 0.92 (0.72-1.18) | 0.74 (0.56-0.97) | 285 | 1.01 (0.46-2.24) | 0.77 (0.33-1.80) |
| Prostate | 18,369 | 0.98 (0.83-1.14) | 0.82 (0.69-0.97) | 1,317 | 0.97 (0.53-1.77) | 1.05 (0.59-1.87) |
| HEMATOLOGICAL CANCER | | |  |  |  |  |
| Leukemia | 2,390 | 0.95 (0.78-1.16) | 0.89 (0.72-1.09) | 308 | 1.45 (0.76-2.78) | 1.41 (0.73-2.74) |
| Myeloma | 1,090 | 1.10 (0.79-1.53) | 1.13 (0.81-1.58) | 119 | 0.77 (0.29-2.06) | 0.91 (0.34-2.43) |
| Hodgkin lymphoma | 947 | 0.86 (0.53-1.42) | 0.72 (0.42-1.22) | 165 | 1.21 (0.40-3.69) | 0.68 (0.16-2.90) |
| Non-Hodgkin lymphoma | 2,891 | 0.81 (0.65-1.01) | 0.81 (0.64-1.02) | 370 | 0.97 (0.54-1.73) | 0.63 (0.32-1.21) |

Analyses adjusted for year of conscription, conscription center, age at conscription, body mass index at conscription, and date of cancer diagnosis. Cardiorespiratory fitness evaluated with maximal aerobic workload and transformed to a standardized score (1-9) and categorized into low CRF (1-5), moderate CRF (6-7), and high CRF (8-9), with low being the reference in the analyses. BMI = Body mass index.

Supplementary table S10. Associations between body mass index in youth and five-year mortality per cancer site for all cancer cases, regardless of previous cancers. Stratified by cardiorespiratory fitness at conscription.

|  | Low CRF | | | | Moderate-high CRF | | | |
| --- | --- | --- | --- | --- | --- | --- | --- | --- |
| Cancer site | **N cases** | **Underweight** | **Overweight** | **Obesity** | **N cases** | **Underweight** | **Overweight** | **Obesity** |
|  |  | **HR (95% CI)** | **HR (95% CI)** | **HR (95% CI)** |  | **HR (95% CI)** | **HR (95% CI)** | **HR (95% CI)** |
| Any cancer site | 28,519 | 0.99 (0.92-1.06) | 1.32 (1.19-1.47) | 1.85 (1.53-2.24) | 56,102 | 1.02 (0.92-1.13) | 1.38 (1.28-1.48) | 1.95 (1.65-2.30) |
| Malignant skin | 8,558 | 0.87 (0.67-1.13) | 1.44 (0.99-2.12) | 1.45 (0.59-3.53) | 19,801 | 1.19 (0.87-1.63) | 1.53 (1.22-1.92) | 2.43 (1.37-4.30) |
| Bronchi and lung | 1,144 | 0.96 (0.81-1.14) | 1.20 (0.84-1.71) | 1.23 (0.74-2.06) | 1,358 | 0.87 (0.64-1.19) | 0.99 (0.79-1.24) | 0.99 (0.58-1.68) |
| Head and neck | 1,358 | 1.00 (0.74-1.35) | 1.64 (1.11-2.42) | 1.76 (0.72-4.33) | 2,191 | 1.35 (0.89-2.04) | 1.39 (1.02-1.90) | 1.09 (0.45-2.64) |
| Central nervous system | 968 | 1.02 (0.81-1.29) | 1.06 (0.77-1.45) | 0.88 (0.45-1.72) | 1,969 | 1.18 (0.88-1.59) | 1.01 (0.81-1.27) | 0.94 (0.47-1.89) |
| Thyroid gland | 270 | 1.58 (0.61-4.04) | 2.17 (0.61-7.73) | NA | 578 | 1.08 (0.26-4.53) | 1.12 (0.50-2.51) | 4.54 (1.78-11.55) |
| GASTROINTESTINAL CANCER | | |  |  |  |  |  |  |
| Esophagus | 424 | 0.98 (0.71-1.36) | 1.20 (0.80-1.81) | 1.22 (0.69-2.15) | 567 | 1.00 (0.56-1.76) | 1.07 (0.81-1.41) | 1.12 (0.58-2.18) |
| Stomach | 496 | 0.81 (0.58-1.12) | 1.03 (0.67-1.60) | 1.68 (0.97-2.92) | 773 | 1.34 (0.88-2.03) | 1.13 (0.87-1.46) | 0.82 (0.39-1.74) |
| Pancreas | 668 | 0.97 (0.77-1.22) | 0.95 (0.67-1.36) | 1.29 (0.57-2.94) | 1,141 | 1.41 (1.02-1.93) | 0.94 (0.76-1.17) | 1.43 (0.84-2.44) |
| Liver, bile ducts and gallbladder | 690 | 1.14 (0.89-1.46) | 1.07 (0.77-1.48) | 1.35 (0.81-2.26) | 883 | 0.92 (0.61-1.40) | 0.93 (0.72-1.21) | 0.99 (0.49-2.00) |
| Colon | 1,592 | 1.19 (0.94-1.51) | 1.11 (0.79-1.55) | 1.12 (0.62-2.01) | 2,673 | 1.02 (0.75-1.40) | 0.95 (0.75-1.20) | 1.19 (0.74-1.90) |
| Rectum | 1,122 | 1.02 (0.75-1.38) | 1.46 (0.98-2.17) | 1.45 (0.64-3.30) | 2,001 | 1.03 (0.68-1.56) | 1.41 (1.06-1.87) | 1.55 (0.85-2.82) |
| UROLOGICAL CANCER | | |  |  |  |  |  |  |
| Kidney | 869 | 0.66 (0.41-1.06) | 1.65 (1.07-2.54) | 1.88 (0.90-3.92) | 1,419 | 0.78 (0.42-1.47) | 1.28 (0.95-1.72) | 1.86 (1.10-3.13) |
| Bladder | 1,162 | 0.73 (0.47-1.12) | 0.74 (0.37-1.47) | 1.65 (0.65-4.19) | 1,916 | 0.88 (0.39-1.99) | 0.93 (0.58-1.52) | 2.14 (0.88-5.21) |
| Prostate | 6,771 | 0.87 (0.67-1.15) | 1.02 (0.59-1.75) | 2.21 (0.70-6.94) | 12,915 | 0.76 (0.48-1.21) | 1.38 (1.05-1.81) | 2.50 (1.33-4.67) |
| HEMATOLOGICAL CANCER | | |  |  |  |  |  |  |
| Leukemia | 861 | 1.32 (0.94-1.85) | 0.63 (0.31-1.25) | 1.30 (0.57-2.98) | 1,837 | 1.51 (0.98-2.33) | 1.08 (0.80-1.47) | 1.33 (0.63-2.80) |
| Myeloma | 378 | 1.77 (0.99-3.16) | 1.49 (0.66-3.35) | 0.95 (0.12-7.24) | 831 | 1.65 (0.80-3.39) | 1.28 (0.80-2.05) | 0.49 (0.07-3.52) |
| Hodgkin lymphoma | 347 | 1.67 (0.71-3.92) | 0.73 (0.27-1.95) | 1.22 (0.28-5.27) | 765 | 1.61 (0.68-3.77) | 0.94 (0.40-2.19) | 4.62 (1.39-15.35) |
| Non-Hodgkin lymphoma | 1,058 | 0.74 (0.48-1.14) | 1.08 (0.67-1.73) | 1.12 (0.41-3.07) | 2,203 | 0.99 (0.55-1.76) | 1.18 (0.83-1.70) | 1.41 (0.69-2.86) |

Analyses adjusted for year of conscription, conscription center, age at conscription, cardiorespiratory fitness at conscription, and date of cancer diagnosis. BMI = Body mass index. Categorized into underweight (<18.5 kg/m2), normal weight (18.5-24.9 kg/m2), overweight (25-29.9 kg/m2), and obesity (≥30 kg/m2). Reference is normal weight.
